# Tripeptides Featuring Dehydrophenylalanine and Homophenylalanine: Homo- Versus Hetero-Chirality and Sequence Effects on Self-Assembly and Gelation

**DOI:** 10.3390/gels11030164

**Published:** 2025-02-24

**Authors:** André F. Carvalho, Teresa Pereira, Carlos Oliveira, Pedro Figueiredo, Alexandra Carvalho, David M. Pereira, Loic Hilliou, Manuel Bañobre-López, Bing Xu, Paula M. T. Ferreira, José A. Martins

**Affiliations:** 1 Center of Chemistry, University of Minho, 4710-057 Braga, Portugalcarlos.oliveira.6b@gmail.com (C.O.); 2 CNC—Center for Neuroscience and Cell Biology, Institute for Interdisciplinary Research (IIIUC), University of Coimbra, 3004-504 Coimbra, Portugal; 3 PhD Programme in Experimental Biology and Biomedicine, Institute for Interdisciplinary Research (IIIUC), University of Coimbra, Casa Costa Alemão, 3030-789 Coimbra, Portugal; 4 Almac Sciences, Department of Biocatalysis and Isotope Chemistry, Almac House, 20 Seagoe Industrial Estate, Craigavon BT63 5QD, UK; 5 REQUIMTE/LAQV, Laboratório de Farmacognosia, Departamento de Química, Faculdade de Farmácia, Universidade do Porto, R. Jorge Viterbo Ferreira, n 228, 4050-313 Porto, Portugal; 6 Institute for Polymers and Composites, University of Minho, 4800-058 Guimarães, Portugal; loic@dep.uminho.pt; 7 International Iberian Nanotechnology Laboratory (INL), Av. Mestre José Veiga s/n, 4715-330 Braga, Portugal; manuel.banobre@inl.int; 8 Department of Chemistry, Brandeis University, 415 South Street, Waltham, MA 02453, USA

**Keywords:** homophenylalanine, dehydrophenylalanine, *N*-succinyldehydrotripeptides, homochiral, heterochiral, self-assembly, hydrogels

## Abstract

Over the years, our research group developed dehydrodipeptides *N*-capped with aromatic moieties as protease-resistant efficacious hydrogelators, affording self-assembled hydrogels at low (critical) concentrations. Dehydrotripeptides, with different dipeptide sequences and (*D*,*L*) stereochemistry, open a wider chemical space for the development of self-assembled soft nanomaterials. In this work, a small library of *N*-succinylated dehydrotripeptides containing a *C*-terminal dehydrophenylalanine (∆Phe) residue and a scrambled dipeptide sequence with phenylalanine (Phe) and homophenylalanine (Hph) (*L*-Phe-*L*,*D*-Hph and *L*,*D*-Hph-*L*-Phe) was synthesized and characterized as a potential hydrogelator. Two pairs of diastereomeric tripeptides were synthesized, both as *C*-protected methyl esters and as deprotected dicarboxylic acids. Peptides with the sequence Hph-Phe-ΔPhe were obtained as a pair (*D*,*L*,*Z*)/(*L*,*L*,*Z*) of diastereomers. Their scrambled sequence analogues Phe-Hph-ΔPhe were obtained also as a diastereomeric (*L*,*D*,*Z*)/(*L*,*L*,*Z*) pair. The effect of stereochemistry (homo- vs. hetero-chirality) and sequence (Phe-∆Phe vs. Hph-∆Phe *motif*) on the self-assembly, biocompatibility, gelation and rheological properties of the hydrogels was studied in this work. Accessible, both as *C*-protected methyl esters and as dicarboxylic acids, *N*-succinylated dehydrotripeptides are interesting molecular architectures for the development of supramolecular nanomaterials. Interestingly, our results do not comply with the well-documented proposition that heterochiral peptides display much higher self-assembly propensity and gelation ability than their homochiral counterparts. Further studies will be necessary to fully understand the interplay between peptide sequence and homo- and hetero-chirality on peptide self-assembly and on the properties of their supramolecular materials.

## 1. Introduction

Supramolecular peptide-based soft nanomaterials are emerging as potential therapeutics, drug delivery nanocarriers, theragnostic platforms and as model systems to study cellular processes relying on self-assembly [1,2,3]. In opposition to classical chemical (bio)synthesis, relying on stepwise construction of strong covalent bonds, self-assembly is driven by cooperative action of a (massive) collection of non-covalent weak intermolecular interactions: hydrophobic and aromatic π-π stacking interactions, electrostatic, polar and hydrogen bonding [4,5]. Nature creates structural diversity and (emerging) functionality through repurposing, combination and self-assembly of a limited pool of building blocks: amino acids, sugars, lipids and nucleobases [6]. Self-assembly is fast, economical and modular. Peptides are privileged monomers for the bottom-up supramolecular assembly of complex nanostructures and architectures. Twenty canonical amino acids, containing side chains with different functional groups, size and shape, polarity and charge can generate an enormous variety of structures. Peptide structural diversity can be further amplified by side chain elaboration, e.g., phosphorylation, glycosylation, etc. Self-assembled peptide-based hydrogels (SAPHs) embody the success of self-assembly as an enabling technology. SAPHs bring together a unique set of properties: high water content, resulting from a self-assembled fibrillar, a highly hydrated porous 3D network that supports cell homing, differentiation, growth and migration; tunable structural, rheological and functional properties through chemical synthesis; and responsiveness to environmental stimuli [7]. Resembling the extracellular matrix (EC), SAPHs are archetypical materials for in vivo applications, as drug delivery [8] and theragnostic platforms and as scaffolds for cell therapies and tissue engineering. Besides biocompatibility, the prime requisite for in vivo applications of SAPHs, it is also essential to match hydrogel’s elasticity to that of target living tissues, while enforcing injectable properties, for minimally invasive hydrogel administration. Creating an enduring EC-like environment, SAPHs promote cell homing, differentiation, growth and migration [9]. However, in time, hydrogels must be degraded and replaced by EC, thus promoting tissue integration and remodeling. Peptides are highly susceptible to proteolysis by endogenous proteases. Premature degradation of peptide pharmaceutics results in short in vivo life-time and often failure in attaining predicted pharmaceutical outcomes [10]. Replacing *L*-amino acids by their unnatural mirror image *D*-amino acids is the simplest, most logical approach to enhance the proteolytic stability of peptides [11]. The incorporation of nonproteinogenic amino acids into peptides, e.g., β-amino acids [12] or α,α-disubstituted amino acids [13], is an effective solution for enhancing peptide stability. In our research endeavors, we (and others) found that *N*-capped dehydropeptides, di- and tripeptides containing deprotected *C*-terminal dehydrophenylalanine (∆Phe), dehydroaminobutyric acid (∆Abu) or dehydroalanine (∆Ala), and a canonical amino acid residue are very effective gelators, affording SAPHs at low (critical) concentrations. Besides enhancing proteolytic stability, the dehydroamino acid residue also limits the conformational freedom of the peptide backbone, enhancing the self-assembly propensity and gelation [14,15]. Structural diversification of the dehydrodipeptides was attained through variation of the dehydroamino acid residue (∆Phe, ∆Abu, ∆Ala,), incorporation of different canonical amino acid and by variation of the nature of the aromatic *N*-capping group (e.g., Naproxen-Npx, carboxybenzyl-Cbz; 2-Naph-2-naphthylacetyl; Naph- naphthaloyl). Bolaamphiphile-type and *bis*-*N*-capped dehydropeptide architectures revealed also powerful gelators [16,17,18,19,20]. Meaningful correlations between dehydropeptide’s structure, self-assembly propensity, gelation ability and rheological properties of the hydrogels were developed from our studies. More recently, we started exploring dehydrotripeptides as building blocks for the construction of supramolecular soft nanomaterials [21]. Dehydrotripeptides, containing an extra (canonical or nonproteinogenic) amino acid allow the exploration of a wider chemical space (different dipeptide sequences) and stereochemical (enantiomeric/diastereomeric relationships) effects on peptide self-assembly, gelation and hydrogels’ properties. In this work, we envisaged that methyl ester *C*-protected dehydrotripeptides could be rendered water soluble, for pH drop-triggered gelation, by deploying a succinic acid *N*-terminal capping group, instead of conventional aromatic *N*-capping. Moreover, *C*-deprotection by saponification creates dicarboxylic acid analogues of the *C*-protected dehydrotripeptide hydrogelators. Inspired by the exceptional self-assembly propensity of the diphenylalanine motif (Phe-Phe), we assembled a small library of *N*-succinylated dehydrotripeptides containing a *C*-terminal ∆Phe residue and a scrambled dipeptide sequence (*L*-Phe-*L*,*D*-Hph and *L*,*D*-Hph-*L*-Phe) [22,23,24]. Changing the configuration (*D* or *L*) and the position of the homophenylalanine (Hph) residue generated two pairs of distereomeric hydrogelators. The *N*-succinylated dehydrotripeptides were fully characterized regarding self-assembly, gelation and cytotoxicity.

## 2. Results and Discussion

### 2.1. Synthesis

**Scheme 1:**
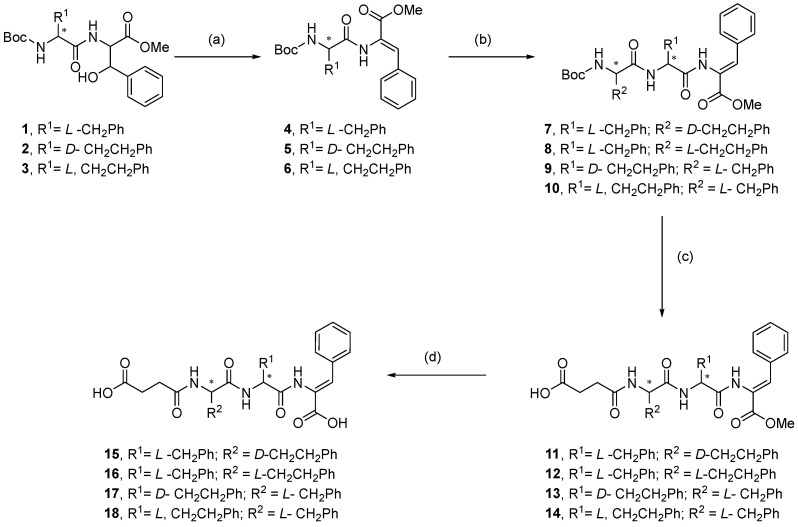
Synthetic pathway for *N*-succinyl dehydrotripeptides **11**–**18**. (a) (i) Boc_2_O, DMAP, dry MeCN, (ii) TMG; (b) Boc-AA-OH, HBTU, Et_3_N, MeCN; (c) (i) TFA, (ii) succinic anhydride, pyridine, N_2_ atmosphere; (d) (i) NaOH (1 M), 1,4-dioxane, (ii) HCl (1 M). TMG = 1,1,3,3-Tetramethyguanidine; HBTU = Hexafluorophosphate Benzotriazole Tetramethyl Uronium.

**Scheme 2:**
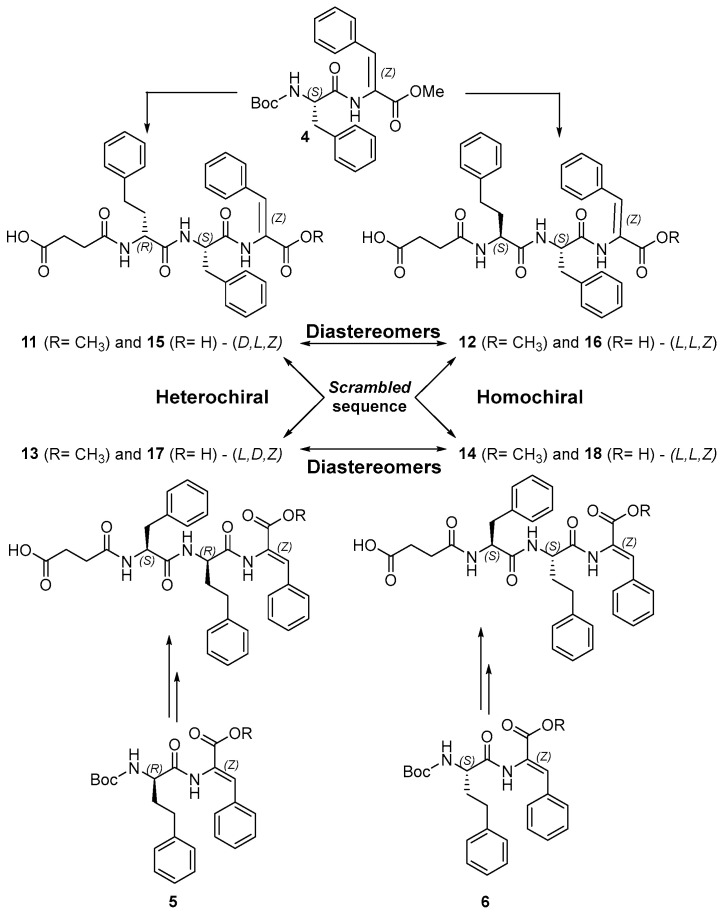
Summary of the stereochemical relationship between dehydrotripeptides **11**–**18**.

A small library of *N*-succinylated dehydrotripeptides was synthesized by the *Boc* solution methodology, reported previously by the research group (Scheme 1) [15]. *Boc*-protected dehydrodipeptide **4**, featuring the *L*-Phe-Z-∆Phe *motif*, and enantiomeric dehydrodipeptides **5** and **6**, containing the *L-* and *D*-Hph-Z-∆Phe *motif*, respectively, were designed as entry points for structural and stereochemical library diversification. Synthons **4**, **5** and **6** were prepared by a one pot two steps dehydration procedure disclosed before: treatment of the corresponding Boc-protected β-hydroxydipeptides **1**–**3** with (Boc)_2_O/DMAP followed by TMG [25]. Starting β-hydroxydipeptides **1**–**3** were prepared under standard HBTU/TEA amide linkage conditions. Following TFA deprotection, dehydrodipeptide **4** was elongated with either, *D*- or *L*-*Boc*-protected Hph, giving direct access to the pair of diastereomeric dehydrotripepides **7** and **8** (*D*,*L*,*Z* and *L*,*L*,*Z*, respectively) with the sequence Hph-Phe-ΔPhe. Elongation of dehydrodipeptides **5** and **6** with *L*-Phe afforded the pair of diastereomeric tripeptides **9** and **10** (*L*,*D*,*Z* and *L*,*L*,*Z*, respectively) featuring the scrambled sequence Phe-Hph-Z-∆Phe (Scheme 2). *Boc*-deprotection (TFA) of compounds **7**–**10**, followed by reaction with succinic anhydride in dry pyridine afforded *N*-succinylated dehydrotripeptides **11**–**14** as *C*-protected methyl esters. Alkaline hydrolysis of the *C*-terminal methyl esters **11**–**14** afforded their dicarboxylic acid analogues **15**–**18**.

This stereochemical and structural diversification strategy afforded a set of *N*-succinylated *C*-protected dehydrotripeptides (**11**–**14**) and a corresponding series of dicarboxylic acids (**15**–**18**) with the same stereochemical configuration (Scheme 2).

**Figure 1:**
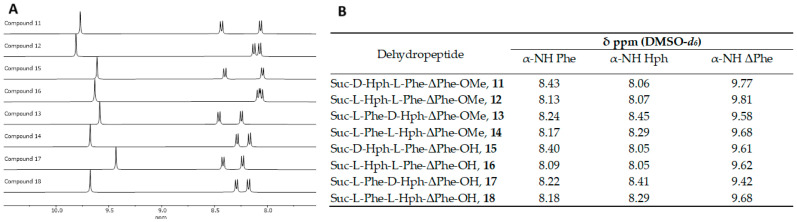
(**A**) Detail of the ^1^H NMR (400 MHz, DMSO-*d*_6_) amide region, α-NH amide backbone protons, for dehydropeptides **11**–**18**. (**B**) Table with characteristic ^1^H NMR signals for the α-NH protons of dehydrotripeptides **11**–**18**.

Distinctive ^1^H NMR (400 MHz, DMSO-*d_6_*) spectral features, chemical shifts and signal multiplicity were observed for the α-NH amide backbone protons of the dehydrotripeptides (Figure 1).

**Figure 2:**
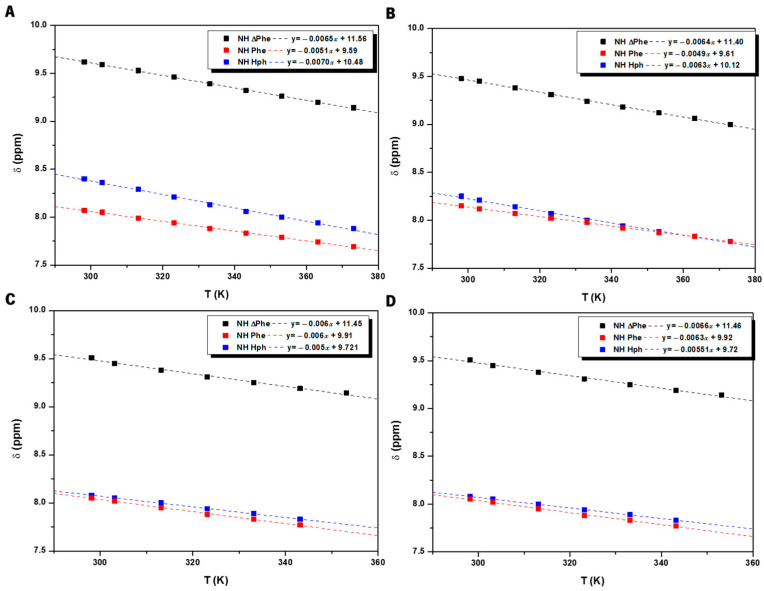
Variable temperature ^1^H-NMR analysis for the α-NH backbone amide signals for peptides: (**A**) **17**; (**B**) **18** in DMSO-*d_6_* and (**C**) **17**; (**D**) **18** in DMSO-*d*_6_ containing 10% *v*/*v* D_2_O.

The signals assigned to the ∆Phe α-NH amide, as singlets, display characteristically high chemical shift, significantly higher than those attributed to the α-NH amide protons of Phe and Hph, presumably owing to conjugation with the α, β-double bond. The ∆Phe α-NH amide protons of the *C*-protected diastereomeric pair **11**/**12**, containing the Phe-ΔPhe *motif*, display the highest chemical shift. *C*-deprotection of the diastereomeric pairs **11**/**12**→**15/16** and **13**→**17** results in significant down field shift of the ΔPhe α-NH resonance, owing to higher electron withdrawing effects of the carboxylic acid group. It is noteworthy that for the diastereomeric dehydropeptide pairs **11**/**12** and **15**/**16**, containing the *motif* Phe-ΔPhe, the heterochiral (*D*,*L*,*Z*) diastereomers **11** (methyl ester) and **15** (dicarboxylic acid) display a similar pattern of signals for the α-NH amide of the Phe and Hph residues, different from that displayed by the homochiral (*L*,*L*,*Z*) **12**/**16** pair. The heterochiral ester/dicarboxylic acid **13**/**17** pair displays also a similar pattern of resonances for the α-NH Phe and Hph residues, different from that exhibited by the homochiral pair **14**/**18**. This result suggests that the peptide backbone of the hetero-chiral and homo-chiral peptides sample different conformations in the non-aggregating solvent DMSO. Owing to higher solubility in DMSO and DMSO/water mixtures, dicarboxylic acid peptides **15**, **16**, **17**, and **18** were selected for variable-temperature ^1^H NMR spectroscopy studies (Figure 2 and Figure S1 and Tables S1 and S2 Supporting Information) [26].

The chemical shift temperature gradients ∆δ/∆T indicate that the NH backbone amide groups are not likely to be involved in intramolecular hydrogen bonds, such as α-helical secondary structures. Significantly, addition of 10% water (*v*/*v*) to the DMSO solvent does not induce any significant change of the amides chemical shift temperature gradients ∆δ/∆T, suggesting that the backbone amides remain exposed to the solvent and do not experience significant structural or environmental changes upon water addition [26]. Nevertheless, these results in (aprotic) DMSO must be interpreted with caution. The stabilizing intra- and intermolecular native hydrogen bonding network of peptides in (protic) water is likely to be very different from that seen in DMSO.

### 2.2. Molecular Dynamics Simulations

**Figure 3:**
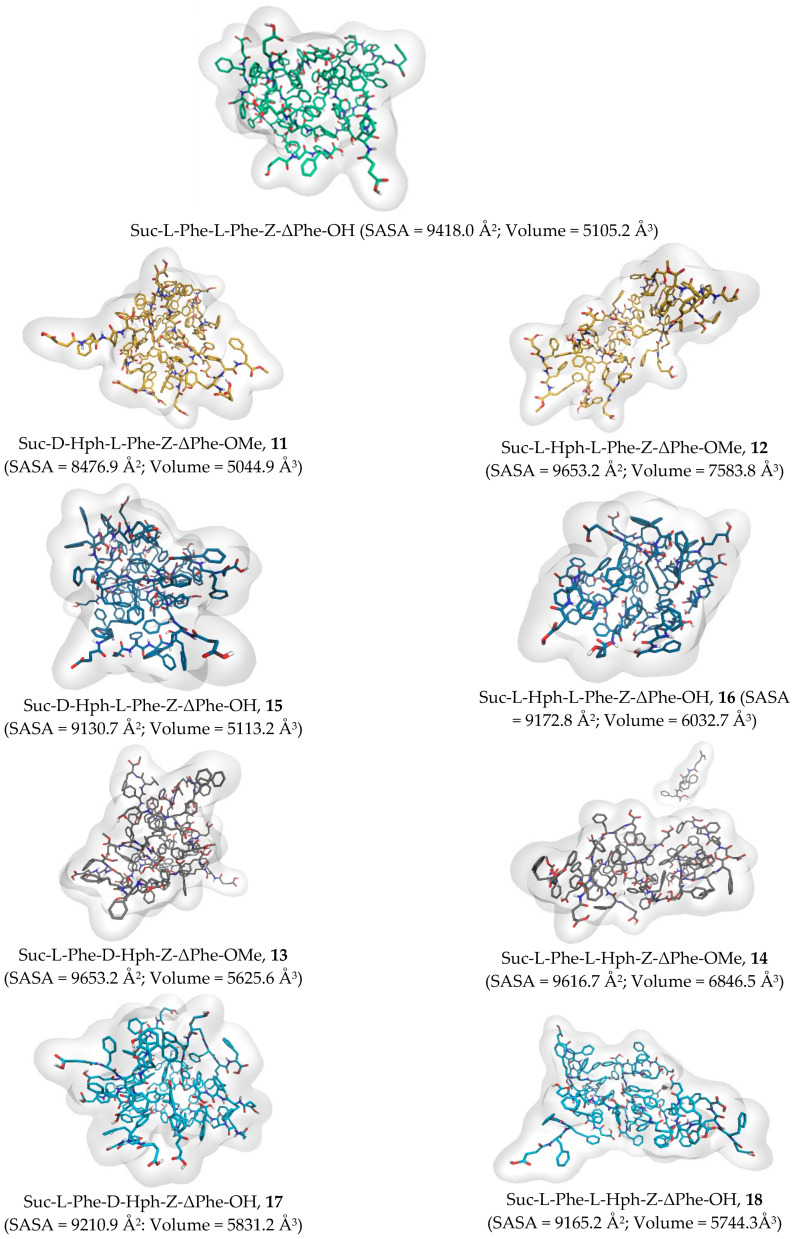
Self-assembled cluster structures obtained by MDS for dehydropeptides **11**–**18**. The parameters SASA and volume are indicated (inside parenthesis). Dehydropeptide Suc-L-Phe-L-Phe-Z-ΔPhe-OH was included in the simulation for control purposes.

Atomistic Molecular Dynamics (MD) simulations were carried out to investigate the self-assembly dynamics, formation of aggregates, and the pattern of representative noncovalent interactions between dehydrotripeptide molecules. A total number of 15 solvated peptide molecules was randomly distributed within an orthorhombic water box. The system was subjected to minimization, followed by heating and equilibration, and was left to run for 250 ns under an isothermal–isobaric ensemble. The randomly dispersed peptide ensembles evolved during the simulation time into large single molecular clusters (Figure 3). The Solvent Accessible Surface Area (SASA) and volume of the self-assembled peptide aggregates was calculated as aggregation propensity indicators (Figure 3 and Supporting Information Figure S2).

**Figure 4:**
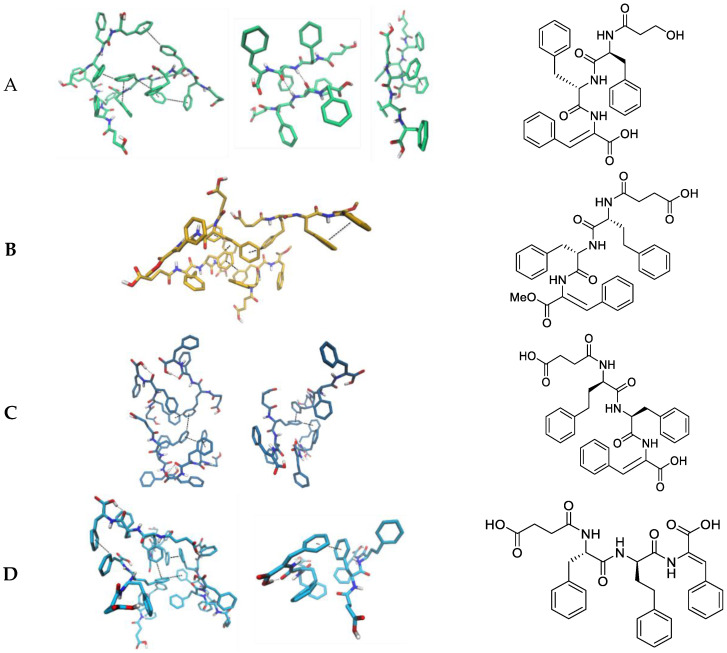
Selected representative intermolecular non-covalent interactions found in peptide aggregates: Suc-L-Phe-L-Phe-Z-ΔPhe-OH (**A**), Suc-D-Hph-L-Phe-Z-ΔPhe-OMe (**11**) (**B**), Suc-D-Hph-L-Phe-Z-ΔPhe-OH (**15**) (**C**) and Suc-L-Phe-D-Hph-Z-ΔPhe-OH (**17**) (**D**). π-interactions are represented as dashed lines. Hydrogen bonds are represented as dotted lines.

With the exception of peptide **11**, originating the most compact aggregate, the peptide aggregates formed by *C*-protected peptides **12**–**14** display higher SASA and volume than their deprotected dicarboxylic acid analogues **16**–**18**, indicating less compactness and more exposed structures, likely resulting from solvent exposure of the methyl ester group. The heterochiral peptides **11** and **15** (*D*,*L*,*Z*), containing the Phe-ΔPhe motif, display lower SASA and volume than the homochiral **12** and **16** (*L*,*L*,*Z*) diastereomers, suggesting more compact aggregates, i.e., higher self-assembly propensity. Moreover, the heterochiral peptide **13** (*L*,*D*,*Z*), containing the Hph-ΔPhe motif, also displays lower SASA and volume than the homochiral diastereomer **14** (*L*,*L*,*Z*). Snapshots of the stable peptide aggregates give insight into the pattern of intermolecular interactions responsible for peptide self-assembly. In the aggregates of peptide Suc-L-Phe-L-Phe-Z-ΔPhe-OH there are some intermolecular hydrogen bonds, between the backbone amide groups and the terminal carboxylic acid, and T-shaped π aromatic stacking interactions, more frequent that parallel interactions (Figure 4A). In the aggregates of the heterochiral dicarboxylic acid **15** (*D*,*L*,*Z*), containing the Phe-ΔPhe motif, the introduction of the Hph residue, results in fewer intermolecular hydrogen bonds. The *C*-terminal carboxylic acid group engages in intramolecular hydrogen bonding with the Phe backbone amide owing to close spatial proximity. The aromatic rings of the Hph residue establish an intermolecular network (clusters) of T-shaped π-π stacking interactions. The Phe aromatic residues engage predominantly in intramolecular T-shaped π-π stacking interactions (Figure 4C). In the aggregates of peptide **11**, the *C*-protected counterpart of dicarboxylic acid **15**, the ester group predominantly faces the solvent. The aromatic residues, especially the Hph, establish a cluster of π-π stacking interactions similar to that seen for peptide **15** (Figure 4B). The aggregates of the dicarboxylic acid heterochiral peptide **17** (*L*,*D*,*Z*) containing the Hph-ΔPhe *motif*, display predominantly clusters between the Hph side chains, via T-shaped π-π stacking interactions, and further intermolecular aromatic stacking interactions between the Hph and Phe and Phe Phe residues. The aggregates of ester **13** show a pattern of intermolecular interactions very similar to those described for peptide **11**, with the methyl ester group facing the solvent. Interestingly, the aggregates of the homochiral peptides **16** and **18,** containing the Hph-ΔPhe *motif*, seem to form fewer clusters between the Hph rings than the aggregates of peptides **15** and **17,** but a higher number of intramolecular Hph-Phe interactions resulting in the transition from a compact spherical aggregate shape (for peptide aggregates **15**/**17**) to a more elongated less compact shape for peptide aggregates **16**/**18**. Regarding the effect of the *C*-terminal on the overall array of intermolecular interactions, it seems that in the C-protected peptides the ester group predominantly faces the solvent. For the deprotected dicarboxylic acid peptides the *C*-terminal carboxylic acid stays in a more balanced position facing both the external zone of the aggregate as well as the inside, where some hydrogen bonding was observed. Overall, the MD simulations suggest that the self-assembly of the dehydrotripeptides is mostly driven by an intermolecular network (clusters) of T-shaped π-π stacking interactions. Hydrogen bonding seems to play a minor role in self-assembly, as indicated also by the NMR variable temperature experiments. Establishing a stronger network of clusters between the Hph side chains, the heterochiral peptides are likely to display higher self-assembly propensity.

### 2.3. Hydrogelation

**Figure 5:**
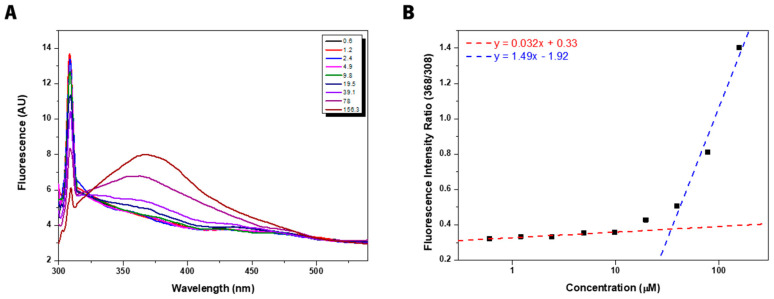
Illustrative example, for dehydropeptide **11,** of the procedure used for determination of the critical aggregation concentration (CAC) for dehydrotripeptides **11**–**18**: (**A**) Steady-state fluorescence spectra (λ_exc_ = 280 nm) for dehydropeptide **11** in the concentration range 0.6 to 156 μM; (**B**) semilogarithmic graphical representation of the concentration dependence of the fluorescence intensity ratio (I_368_/I_308_).

The Critical Aggregation Concentration (CAC) was determined for dehydrotripeptides **11**–**18** from the concentration-dependence of the fluorescence intensity emission ratio at 368 nm (aggregates) and 308 nm (monomer) (I_368_/I_308_) (Figure 5 and Table 1) (Figure S2 Supporting Information) [27].

The CAC values determined for hydrogelators **11**–**18** are of the same order of magnitude (Table 1), ranging from 33 to 41 μM, indicating comparable self-assembly propensity. The higher hydrophobicity of the ester protected peptides **11**–**14** does not translate into higher self-assembly propensity comparing to their less hydrophobic dicarboxylic acid counterparts **15**–**18**. The stereochemistry of the peptides seems nonetheless to have a slight effect on the peptide’s aggregation propensity, with heterochiral peptides **11**, **13**, **15** and **17** displaying slightly lower CAC than their homochiral analogues, in qualitative agreement with the measured SASA values.

**Figure 6:**
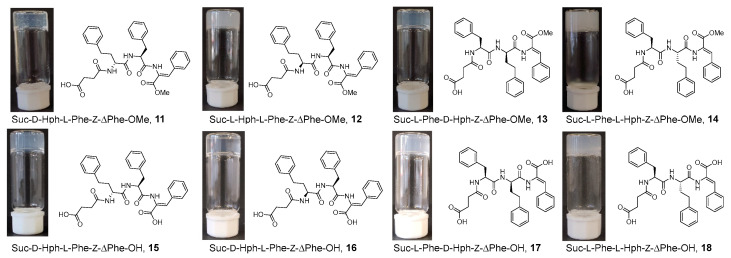
Images of the *N*-succinyl dehydrotripeptides hydrogels **11**–**18**.

The hydrogelation ability of the *N*-succinyl dehydrotripeptides was tested by the pH-trigger approach, by addition of glucono-δ-lactone (GdL). Initially, peptide suspensions in water were made soluble, by addition of diluted aqueous NaOH until alkaline pH (9–10). Gelation was triggered by GdL. It is worth noting that the methyl ester *C*-protected peptides **11**–**14** are made water soluble by ionization of the succinic acid moiety. Compounds **11**–**18** successfully formed self-supporting hydrogels at low critical gelation concentration (CGC) 0.2 wt% (3.4–3.5 mM) (Figure 6, Table S3 Supporting Information). The gels reached a final pH in the range 4.5–5.5 suggesting that gelation is triggered by (partial) protonation of the carboxylic acid groups. The homochiral and heterochiral peptides show identical gelation ability. Similar CAC values seem to translate into equivalent gelation ability, neither sequence nor diastereomeric configuration dependent. Previous studies with low molecular weight peptides indicate that heterochiral peptides display enhanced self-assembly and gelation capabilities, originating more stable hydrogel networks than their homochiral counterparts [28,29,30,31]. Recently Marchesan et al. [32] studied the gelation ability of fully deprotected tripeptide diastereomers H-L-Leu-L-Phe-L-Phe-OH (*L*,*L*,*L*) and H-D-Leu-L-Phe-L-Phe-OH (*D*,*L*,*L*) and found that the heterochiral (*D*,*L*,*L*) tripeptide originated a self-supporting hydrogel at pH 7.4 using a pH trigger, but its homochiral (*L*,*L*,*L*) diastereomer showed limited gelation ability, failing to originate a persistent supramolecular gel. As seen in the MDS experiments the self-assembly of the tripeptides seems to be mainly driven by an extensive array of aromatic stacking interactions, likely resulting from the all-aromatic-amino acid nature of the peptides. Conformational restrictions imposed by the dehydroamino acid may also override the effect of sequence and chirality on self-assembly observed for all-canonical amino acid peptides.

### 2.4. Transmission Electron Microscopy

**Figure 7:**
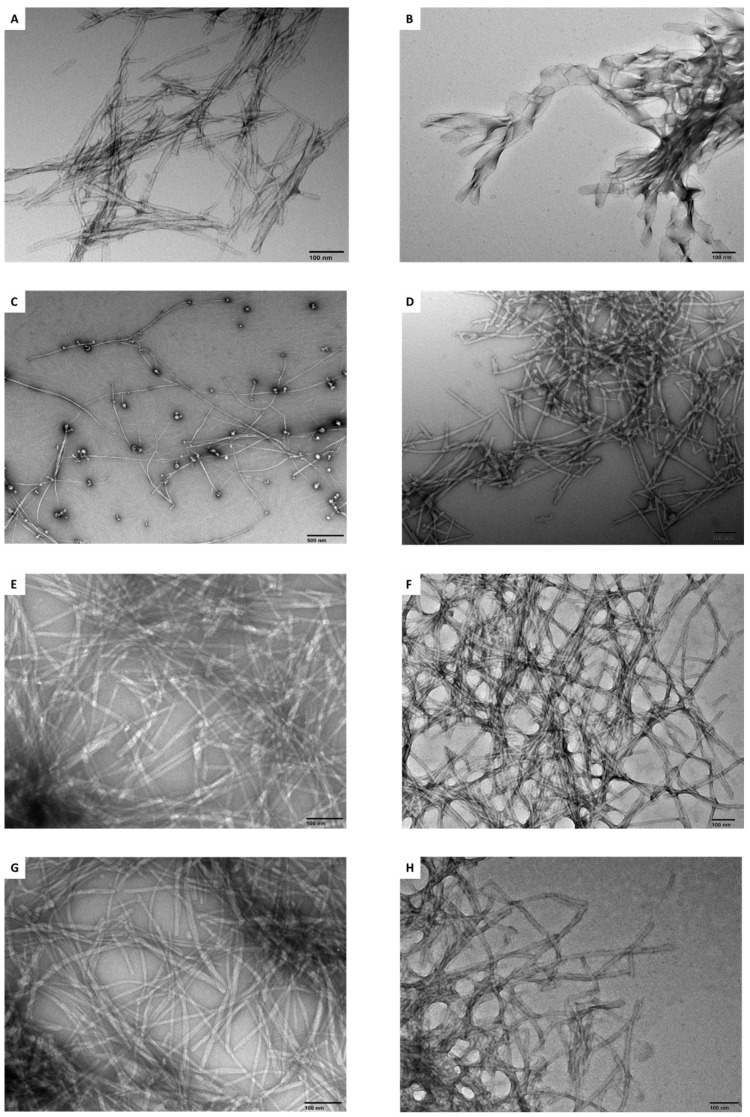
TEM images for hydrogels **11**–**18** (0.6 wt%) ((**A**–**H**) respectively), obtained by the GdL pH dropping methodology.

Transmission electron microscopy (TEM) images were acquired for hydrogels **11**–**18** to get insight into the micro- and nano-structuring of the hydrogel’s matrix (Figure 7). Overall, the hydrogels show fibrillar networks, with variable density, fiber thickness ranging from 10 to 40 nm, and lengths spanning from 500 nm to 3 µm. In general, the hydrogels formed by the dicarboxylic acid hydrogelators **15**–**18** display networks with higher fiber density than their methyl esters analogues **11**–**14**. Interestingly, the TEM images of the hydrogel formed by heterochiral peptide **13** (*L*,*D*,*Z*), featuring the Hph-ΔPhe motif, show long very thin fibers mixed with darker circular structures, likely representing vesicle-type aggregates. In contrast, the TEM images for the homochiral peptide **12**, bearing the Phe-ΔPhe motif, show bundles of thick ribbon-like structures, exhibiting helicity. Amongst the methyl ester hydrogelators **11**–**14**, the homochiral peptide **14** showed the denser fiber network. It is worth noting that homochiral peptides **12** and **14** display denser fiber networks, made of thicker fibers, than their heterochiral analogues **11** and **13**. Moreover, diastereomers pair **11**/**12**, featuring the Phe-ΔPhe motif seems to originate thicker fibers than their analogues **13**/**14** with the Hph-ΔPhe motif. Amongst the dicarboxylic acid dehydropeptides **15**–**18** the TEM images for the homochiral pair **16**/**18** suggest a higher degree of fiber twisting, coiling and cross-linking than the heterochiral couple **15**/**17**. Overall, it seems that both peptide sequence and chirality govern the hydrogels’ network architecture.

### 2.5. Circular Dichroism

CD spectra were acquired for dehydrotripeptides **11**–**18** using peptide concentrations (0.01 wt%, 170–175 µM) below the CGC but well above the *CAC*. GdL (0.01 wt%) was used as pH trigger for peptide self-assembly, mimicking the experimental conditions used for gelation.

**Figure 8:**
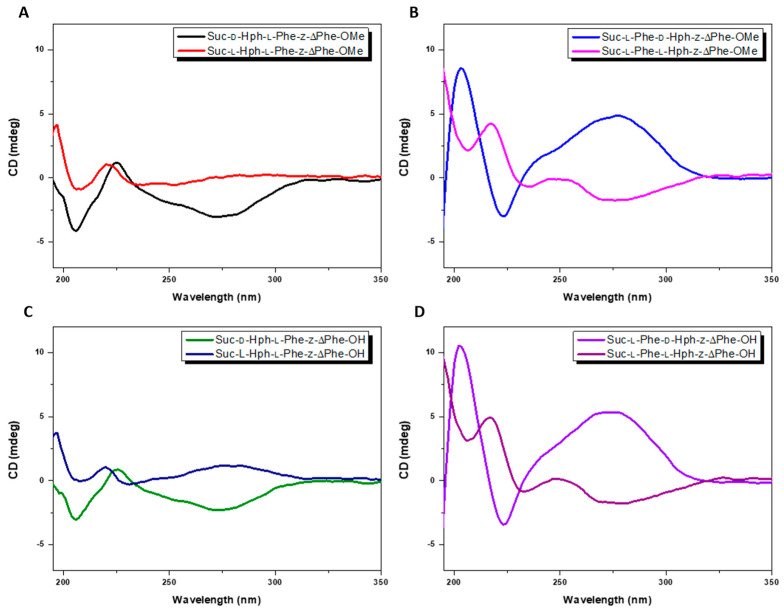
CD spectra for dehydrotripeptides **11**–**18** (0.01 wt%, 170–175 µM). (**A**) Diastereomers **11** and **12**; (**B**) Diastereomers **13** and **14**; (**C**) Diastereomers **15** and **16** and (**D**) Diastereomers **17** and **18**.

The CD spectra of peptides **11**–**18** show a broad band at around 280 nm, assigned to chiral stacking of the aromatic Phe, Hph and ΔPhe residues, as seen in the MDS experiments (Figure 8). Remarkably, this band reveals a mirror-like relationship for the diastereomeric peptide pairs **11**/**12** and **15**/**16**, containing the Phe-ΔPhe motif, and **13**/**14** and **17**/**18**, featuring the Hph-ΔPhe motif. The chiral stacking of the aromatic residues in the self-assembled aggregates seems to be determined by the diastereomeric configuration of the peptides. The heterochiral ester/dicarboxylic acid pair **13**/**17**, containing the Hph-ΔPhe motif, displays the highest intensity suggesting higher order chiral stacking. The secondary structure of the self-assembled fibers seems to be determined by peptide sequence (Table S4 Supporting Information): heterochiral peptides **11** and **15**, containing the motif Phe-ΔPhe, display random coil secondary structure, evidenced by a negative band around 200 nm. In contrast, heterochiral peptides **13** and **17**, featuring the Hph-ΔPhe motif, exhibit β-sheet secondary structure as indicated by the characteristic positive and negative bands at 200 nm and 225 nm, respectively. Interestingly, the homochiral peptide couples **12**/**16** and **14**/**18**, containing L-Hph, display bands around 190 nm and 225 nm, assigned to α-helix and random coil structures, respectively. The peptide sequence, Phe-ΔPhe or Hph-ΔPhe motif, influences significantly the secondary structure of the fibers. In contrast, the stereochemistry of the peptides, with the same sequence, has a less drastic effect in determining the secondary structure of the self-assembled fibers.

### 2.6. Rheology

**Figure 9:**
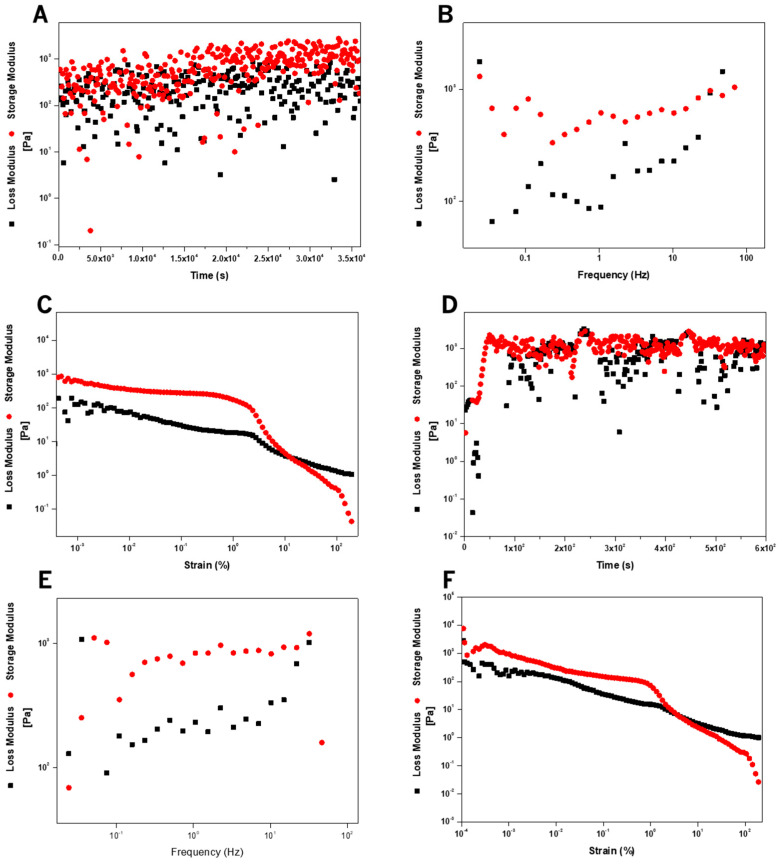
Rheological procedure deployed for characterization of hydrogels **11**–**18,** illustrated here for hydrogel **11** (0.2 wt%): (**A**) Kinetic study at constant frequency (*f* = 1 Hz) and strain (1 × 10^−4^%); (**B**) Mechanical frequency sweep, at constant strain (1 × 10^−4^%); (**C**) Large amplitude oscillatory shear (strain sweep) at constant frequency (*f* = 1 Hz); (**D**) Hydrogel reformation kinetic study at constant frequency (f = 1 Hz) and strain (1 × 10^−4^%); (**E**) Mechanical frequency sweep of reformed hydrogel at constant strain (1 × 10^−3^%); (**F**) Large amplitude oscillatory shear (strain sweep) for reformed hydrogel at constant frequency (*f* = 1 Hz).

A rheological methodology was designed to study the structural evolution and recovery behavior of the hydrogels formed by dehydrotripeptides **11**–**18** (Figure 9). First, the formation kinetics of the hydrogels was studied by recording the time-dependent evolution of the storage G′ and loss G″ moduli, at constant deformation, until reaching a stable plateau, corresponding to formation of a stable hydrogel network (Figure 9A). Next, a frequency sweep test was performed (at constant 1 × 10^−4^% strain) to evaluate the frequency dependence of G′ and G″ and the structural stability of the hydrogel network (Figure 9B). After this, a large amplitude oscillatory shear (LAOS) experiment was performed to evaluate the hydrogel’s critical yielding strain, beyond which hydrogels began to break, measuring the hydrogel’s mechanical robustness upon increasing deformation (Figure 9C). The same set of rheological testes was further carried out to study the self-healing properties of the mechanically broken hydrogels (Figure 9D,E and Figures S3–S17 Supporting Information).

**Figure 10:**
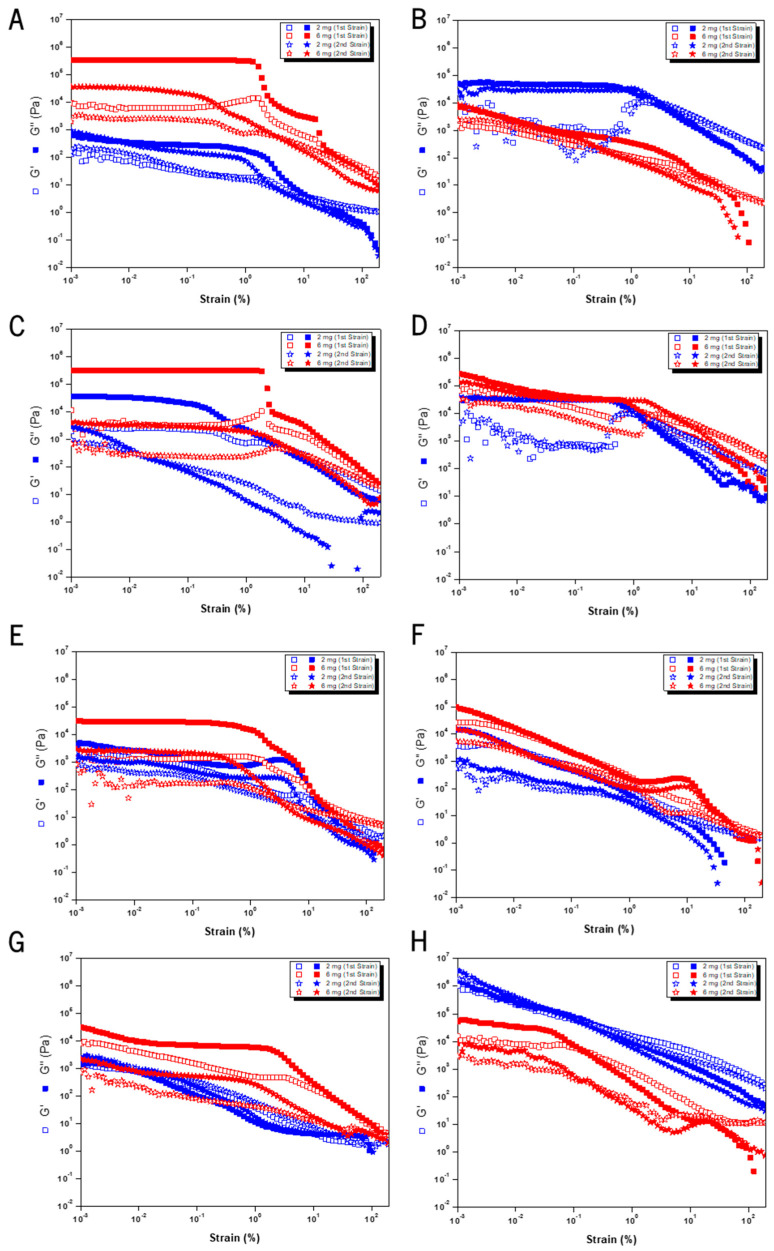
Strain sweep experiments at constant frequency, *f* = 1 Hz (solid symbols: G′; empty symbols: G″; blue: 0.2 wt%; red: 0.6 wt%) for hydrogels **11**–**18** ((**A**) compound **11**, (**B**) compound **12**, (**C**) compound **13**, (**D**) compound **14**, (**E**) compound **15**, (**F**) compound **16**, (**G**) compound **17**, (**H**) compound **18**) before and after breaking.

Figure 10 illustrates the strain dependence of the storage (G′) and loss (G″) moduli for hydrogels **11**–**18** at 0.2 and 0.6 wt%, before and after reformation.

Hydrogels **11**–**18** were characterized at 0.2 wt% concentration, corresponding to the empirical *CGC* value, and at 0.6 wt%, well above the *CGC*, to ensure full formation of the hydrogel’s fibrillar network. At both concentrations, 0.2 and 0.6 wt%, at low strain, G′ is higher than G″ and the ratio G′/G″ is around 10, indicating a dominant elastic behavior for all hydrogels. Moreover, the majority of the 0.6 wt% hydrogels display, around an order of magnitude, higher elasticity (G′) than the 0.2 wt% gels. Higher peptide concentrations are likely to result in fibril networks with higher density and entanglement crosslinking extent. Hydrogels **12** and **18** are exceptions to this trend: the 0.2 wt% hydrogels show around an order of magnitude higher elasticity than the 0.6 wt% hydrogels. The TEM images obtained at 0.6 wt%, reveal that hydrogels **12** and **18** are made of thicker fibers, with lower extent of entanglement, comparing to the other hydrogels (Figure 7). For most hydrogels there is a region of linear elasticity and viscosity strain dependence, between 0.001–1%. Further increasing strain results in a reduction of both G′ and G″. Eventually, a crossover point is reached, indicating transition from elastic (gel) to viscous (liquid-like) behavior marking gel breakup (yielding), associated to breakdown of the hydrogel network. Hydrogel **11** shows an early crossover of G′ and G″, especially the 0.6 wt% gel, indicating that it is more prone to softening under strain (Figure 10A). Gel **12** exhibits delayed crossover and maintains higher G′ values over a wider strain range, suggesting a stronger gel structure at both concentrations (Figure 10B). Hydrogels **13** (Figure 10C) and **14** (Figure 10D) display similar trends, with hydrogel **13** showing slightly higher initial G′ values compared to **14**. Hydrogels **15** (Figure 10E) and **16** (Figure 10F) have high G′ values at lower strains, but compound **16** shows a sharp drop in G′ after the crossover point, indicating easier network breakdown at higher strains. Finally, gels **17** (Figure 10G) and **18** (Figure 10H) show strong gel-like behavior, with hydrogel **17** showing more pronounced strain softening than hydrogel **18**, suggesting a weaker network structure for hydrogel **17**. Hydrogel **17** weaker network structure (as evidenced in the TEM images by less twisting and cross-linking extent) reasonably explains its pronounced strain softening compared to the more robust network of hydrogel **18**. Regarding gels’ elasticity (G′), with the exception of peptide **12**, the *C*-protected peptides (**11**–**14**), originate hydrogels displaying higher elasticity, by around one order of magnitude, than their dicarboxylic acid counterparts (**15**–**18**), presumably owing to higher hydrophobicity (Table 2). Accordingly, the TEM images reveal that, in general, the methyl ester hydrogels **11**–**14** form significantly denser fiber networks than their corresponding dicarboxylic acid derivatives **15**–**18** (Figure 7). The distinctive network of hydrogel **12**, made of thick ribbon like nanostructures, may result in reduced entanglement and low elasticity. Interestingly, in the methyl ester peptide’s series **11**–**14**, neither the peptide sequence, Phe-ΔPhe vs. Hph-ΔPhe, nor the configuration (*D* vs. *L*) of the Hph residue, seem to have a significant impact on the hydrogels’ elasticity. The same general trend is also observed for dicarboxylic acid gels **15**–**18**. In general, all hydrogels recover to some extent following mechanical break up. At 0.6 wt%, only the homo-chiral *C*-protected hydrogels **12** and **14** show higher recovery. Most gels, show little recovery (<10% from initial value), presumably as result of incomplete reformation of the fibrillar 3D network. In contrast, at 0.2 wt% most hydrogels show higher recovery (>30% from initial value). The homochiral *C*-protected/dicarboxylic acid hydrogel’s pair **14** and **18,** shows a substantial increase in elasticity upon reformation, suggesting injectable properties suitable for in vivo applications.

### 2.7. Biological Assays

**Figure 11:**
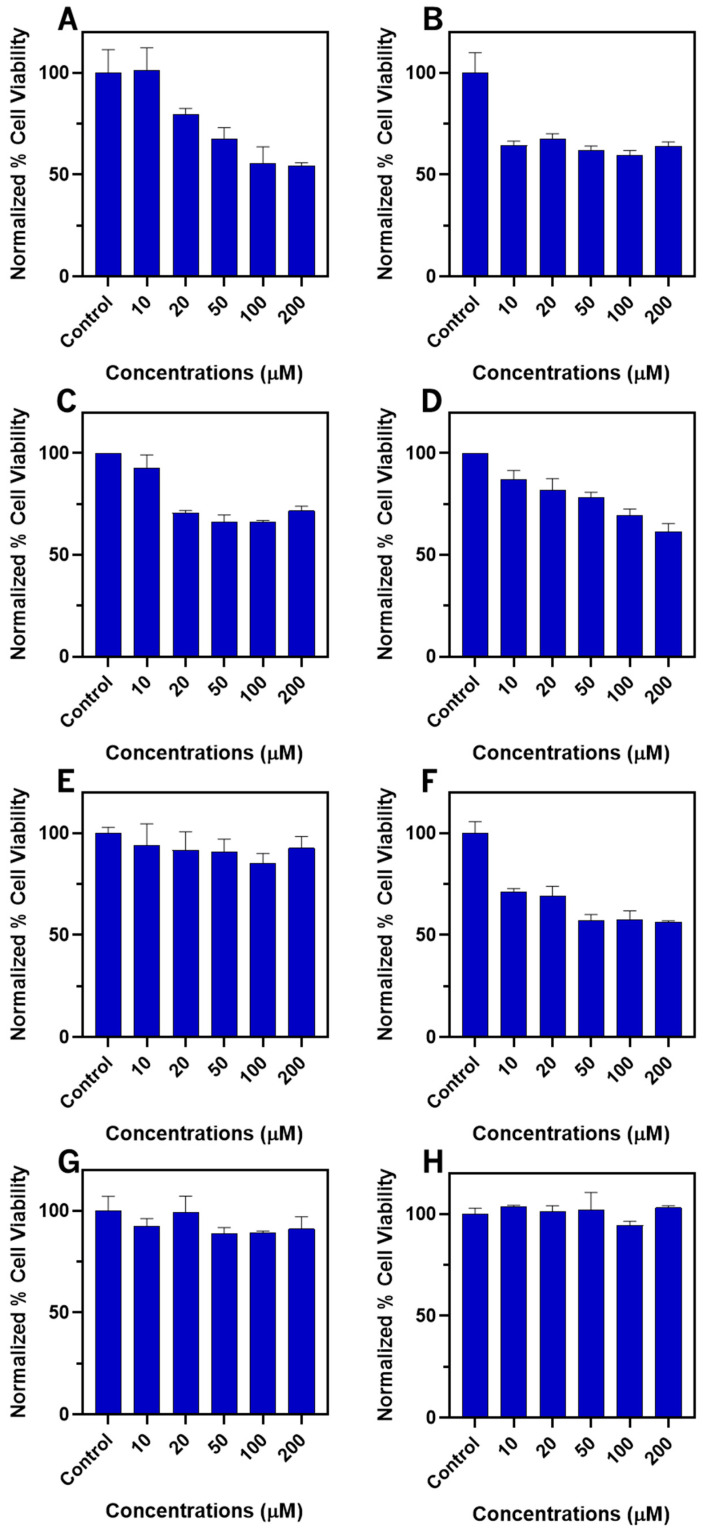
MTT evaluation of the impact of dehydrotripeptides **11**–**18** (**A**–**H**), in the concentration range 10–200 µM, over 24 h incubation period, towards the viability of non-cancerous human bone marrow stromal cell line (HS-5).

Hydrogelator biocompatibility is a fundamental requisite for biological applications, e.g., as drug delivery nanocarriers and therapeutic theragnostic platforms. The safety profile of hydrogelators **11**–**18**, regarding potential biological applications, was evaluated with the non-cancerous human bone marrow stromal cell line (HS-5) by the MTT assay (Figure 11) [33].

The main trend emerging from the MTT assays points to higher toxicity of the C-protected hydrogelators **11**–**14** comparing to their dicarboxylic acid counterparts **15**–**18**. Amongst the methyl esters (**11**–**14**), dehydropeptides **12** and **13** show concentration-independent cytotoxicity, while peptides **11** and **14** display concentration dependent cytotoxicity. Concentration-dependent cytotoxicity suggests interaction with a molecular target. The methyl esters, displaying higher hydrophobicity than their counterpart dicarboxylic acids, are likely to be internalized by cells in higher extent than the dicarboxylic acids. Toxicity mechanisms linked to membrane disruption are also more likely for the amphiphile-like methyl ester dehydropeptides **11**–**14**. The methyl ester/dicarboxylic acid couple **12**/**16**, bearing the Phe-ΔPhe *motif* and the *L*-Hph enantiomer, revealed the most toxic compounds. Moreover, dehydropeptides **12** and **16** display essentially concentration-independent cell toxicity, suggesting a physical cell death mechanism. Interestingly, cell toxicity starts at concentrations (10 µM) bellow the dehydropeptides CAC (40.74 and 38.64 µM, for dehydropeptides **12** and **16**, respectively) excluding toxicity from aggregates. Dehydropeptides **15**, **17** and **18** are essentially non-toxic in the concentration range 10-200 µM. As the dicarboxylic acids display CAC values of the order of magnitude 30–40 µM, both the non-aggregated (monomeric) and aggregated peptide dicarboxylic acid forms are essential nontoxic. Therefore, the dicarboxylic acid-based hydrogels **15**, **17** and **18** are potentially suitable nanocarriers for drug delivery applications. Overall, at the lower concentration range tested, 10 µM and 20 µM, most dehydropeptides did not show a significant effect on the cell viability.

## 3. Conclusions

In this work we synthesized a focused library of succinic acid *N*-capped dehydrotripeptides featuring a *C*-terminal dehydrophenylalanine (∆-Phe) residue and a scrambled dipeptide sequence (*L*-Phe-*L*,*D*-Hph-ΔPhe and *L*,*D*-Hph-*L*-Phe-ΔPhe). Changing the configuration (*L* or *D*) and swapping the position of the Hph residue afforded initially two pairs of methyl ester-protected diastereomers. *N*-succinilated *C*-protected tripeptides formally display inversion of polarity comparing to conventional *C*-deprotected peptides *N*-capped with (nonionizable) aromatic groups. Besides promoting solubility, for gelation purposes, saponification of the *C*-protected peptides generated a subset of dicarboxylic acid peptides with the same sequence and stereochemistry as their *C*-protected precursors. Peptides with the sequence Hph-Phe-ΔPhe **11**/**15** (*D*,*L*,*Z*) and **12**/**16** (*L*,*L*,*Z*) were obtained as a pair of diastereomers. Their *scrambled* sequence analogues Phe-Hph-ΔPhe **13**/**17** (*L*,*D*,*Z*) and **14**/**18** (*L*,*L*,*Z*) were obtained also as a diastereomeric pair. Both the ***C***-protected and the dicarboxylic acid peptides showed similar, sequence and stereochemistry-independent gelation ability, affording hydrogels at low concentrations, 0.2 wt%, using the GdL pH dropping methodology and the empirical inversion tube test. Similar critical aggregation concentrations CAC values were determined for the dehydrotripeptides, suggesting equivalent self-assembly propensity. MD simulations indicated that the self-assembly of the dehydrotripeptides is mostly driven by an intermolecular network (clusters) of T-shaped π-π stacking interactions, predominantly between Hph residues. Hydrogen bonding seems to play a minor role in the self-assembly of the dehydrotripeptides, as indicated also by the NMR variable temperature experiments. The CD study confirms a variable degree of chiral π-π stacking of aromatic sides chains for all dehydropeptides. The slightly higher self-assembly propensity, seen in the self-assembly studies, for the heterochiral peptides, does not translate into strikingly higher gelation ability, as reported in the literature for diastereomeric all-canonical di- and tri-peptides. TEM images of the hydrogels show fibrillar networks, with variable fiber thickness, density and entanglement. Overall, the dicarboxylic acid hydrogelators display networks with higher fiber density than their methyl esters analogues. Both peptide sequence and stereochemistry seem to govern the hydrogels’ network architecture. Regarding gels’ elasticity *C*-protected peptides originate hydrogels displaying significantly higher elasticity than their dicarboxylic acid counterparts, in accordance with the TEM images, showing denser fiber networks. All gels display self-healing properties, lending themselves to biological applications relying on non-invasive administration. In general, the *C*-protected hydrogelators display higher toxicity than their dicarboxylic acid counterparts. The peptides bearing the Phe-ΔPhe motif and the *L*-Hph enantiomer, are especially toxic, displaying essentially concentration-independent cell toxicity, which suggests a physical cell death mechanism. The heterochiral dicarboxylic acid peptides and the homochiral peptide containing the Hph- ΔPhe motif are essentially non-toxic.

In this work we disclosed a synthetic pathway for *N*-succinylated dehydrotripeptides. Accessible, both as *C*-protected methyl esters and as dicarboxylic acids, *N*-succinylated dehydrotripeptides are interesting molecular architectures for development of supramolecular nanomaterials. Interestingly, our results do not comply with the well documented proposition that heterochiral peptides display much higher self-assembly propensity and gelation ability than their homochiral counterparts. Further studies will be necessary to fully understand the interplay of peptide sequence and homo- hetero-chirality on peptide self-assembly and on the properties of their supramolecular materials.

## 4. Materials and Methods

### 4.1. General Procedures

Chemicals, analytical grade reagents and solvents were acquired from Acros (Geel, Belgium) and Sigma-Aldrich (St. Louis, MO, USA) and used as received. Whenever required, solvents were dried by standard methodologies. Aqueous solutions were made using distilled water. Thin Layer Chromatography (TLC), Merck-Kieselgel plates 60 F254, was used to monitor reactions with iodine revelation and examination and under UV. Anhydrous magnesium sulfate (Riedel) was used to dry organic phases.

The ^1^H and ^13^C NMR spectra, elucidated through Distortionless Enhancement by Polarization Transfer (DEPT), Heteronuclear Single Quantum Coherence (HSQC), and Heteronuclear Multiple-Bond Correlation (HMBC) techniques, were acquired utilizing a Bruker Avance III 400 (Billerica, MA, USA) spectrometer. The instrument operated at frequencies of 400.13 MHz and 100.62 MHz for ^1^H and ^13^C NMR, respectively. Spectra were recorded at 25 °C, referencing the residual solvent signals. Deuterated dimethyl sulfoxide (DMSO-*d*_6_) served as the solvent, and chemical shifts are reported in parts per million (ppm) with coupling constants in Hertz (Hz). The elemental analysis was obtained in a LECO instrument (St. Joseph, MI, USA). The mass spectrometry service CACTI, at the University of Vigo, Spain, provided the HRMS data. Mass spectra, Electrospray Ionization (ESI), were acquired with a Thermo Finnigan LxQ (Linear Ion Trap, San Jose, CA, USA) mass spectrometer. All synthesis procedures are described in the Supporting Information.

Fluorescence measurements were conducted at room temperature using a Shimadzu RF5301PC (Kyoto, Japan) spectrometer. The critical aggregation concentration (CAC) of peptides was determined in phosphate-buffered saline (PBS) with a pH = 7.4. The experimental protocol involved peptide solution concentrations ranging from 0.6 to 156 μM. The sample was excited at a wavelength of λ_ex_ = 280 nm and the resulting fluorescence spectrum was recorded within the wavelength range of λ_em_ = 300–550 nm, using excitation and emission bandwidths of 1.5 and 3 nm, respectively. The fluorescence intensity ratio (I_368_/I_308_) was plotted against the logarithm of peptide concentration. The concentration corresponding to the inflection point in the plot was identified as the Critical Aggregation Concentration (CAC).

The molecular structure of compounds **11**–**18** was designed with the USCF Chimera software 1.18 [34], and geometry optimized in Gaussian09 [35] using B3LYP [36] with the 6-31++G(d,p) basis set and the Polarizable Continuum Model solvent description [37]. The atomic partial charges were calculated from the optimized structure resorting to the Restrained Electrostatic Potential (RESP) method [38] from the HF/6-31++G(d,p) single-point energy calculations. For each system, the initial configuration was generated using the PACKMOL program [39], by randomly distributing 15 molecules in a 40 Å cubic box with a distance tolerance of 8 Å. The MD simulations were carried out using compounds **11**–**18** as protonated carboxylic acids. The systems were then solvated with an orthorhombic box with approximately 8500 OPC water molecules [40] using the LEaP module from Amber molecular dynamics program (AMBER18) [41]. No counter ions were required to neutralize the systems.

The GPU-accelerated PMEMD module implemented in AMBER18 [41] with the GAFF2 force field [42] as used to run the Molecular Dynamics simulations. Two initial energy minimizations and 500 ps of equilibration were carried out in an NVT ensemble, using Langevin dynamics with small restraints of 10 kcal mol^−1^ to heat the system. Production simulations were achieved by running 250 ns at 310 K in an NPT ensemble using the Langevin dynamics with a collision frequency of 1 ps^–1^. Constant pressure periodic boundary conditions were imposed with an average pressure of 1 atm. Isotropic position scaling was used to maintain pressure with a relaxation time of 2 ps and the time step was set to 2 fs. SHAKE constraints were applied to all bonds involving hydrogen atoms [43]. The Particle Mesh Ewald method [44] was used to calculate the electrostatic interactions with a cut-off distance of 10 Å. The MD trajectories were centred back to the primary box resorting to the CPPTRAJ [45] module implemented in AMBER18 [41].

The cluster structure representations were acquired through the DBScan clustering algorithm [46] with a minimum of 2 points to form a cluster and 0.7–1.0 Å as cut-off distances concerning the root-mean-square deviation of the Cα. The last 20 ns trajectory of the corresponding atoms was extracted and analyzed using 500 frames for each system.

The volume and SASA of the aggregate of control dehydropeptide Suc-L-Phe-L-Phe-Z-ΔPhe-OH and dehydrotripeptides **11**–**18** were computed using a custom python script executed within the PyMOL [47] environment. To compute the SASA, the built-in get area function was used. The script set the dot solvent and dot density parameters to 1 and 3, respectively, to ensure accurate SASA calculations. The resulting SASA values were stored in the B-factor column of the PDB file. The solvent-excluded volume was then determined by summing these B-factor values, providing a measure of the aggregate’s volume.

### 4.2. Hydrogel Preparation

A 1.0 M NaOH solution was incrementally added to a mixture of hydrogelators (2 mg) in 1.0 mL of water until pH 10 was attained. This mixture underwent sonication for approximately 1 min until complete dissolution, resulting in gelator concentrations of 0.2 wt%. D-glucono-δ-lactone (GdL) (2 or 3 mg) was then added, followed by thorough mixing for 1 min. The solutions were left undisturbed overnight, and the Critical Gelation Concentration (CGC) was determined through tube inversion tests, where gels were identified in tubes exhibiting free-standing material after 5 min of inversion.

### 4.3. Transmission Electron Microscopy (TEM)

Copper grids with a 400-mesh structure, which were coated with a carbon film which were glowed discharged previous usage. Subsequently, sample solutions, each comprising 5 µL, were applied to the grids. After a duration of 60 s, the sample solutions were carefully removed, and the grids underwent staining with a 2% (*v*/*v*) uranyl acetate solution. The stained grids were then allowed to air-dry. Transmission electron microscopy images were acquired using a Morgagni 268 (Hillsboro, OR, USA) transmission electron microscope operating at a high voltage (HV) of 80 kV, with a filament setting of 2.

### 4.4. Circular Dichroism (CD) Spectroscopy

CD spectra were recorded under a constant flow of N_2_ using a Jasco model J-1500 (JASCO, Tokyo, Japan) spectropolarimeter at 25 °C. Solutions of hydrogelators (0.01 wt%) and GdL (0.01 wt%) were loaded into 0.1 mm quartz cells.

### 4.5. Rheological Studies

Gel-forming solutions were injected into the Couette cell of a stress-controlled rotational rheometer (MCR300, Anton Paar GmbH, Graz, Austria). Viscoelastic characterization was performed at 25 °C, and gels underwent dynamic strain sweep testing at 1 Hz up to 100% to assess gel break-up.

### 4.6. Cell Culture and MTT Assay

The human marrow stromal (HS-5) cell line was obtained from the American Type Culture Collection (ATCC, Manassas, VA, USA). Cells were cultured in Dulbecco’s Modified Eagle’s Medium (DMEM) supplemented with 10% fetal bovine serum (FBS), 100 U/mL penicillin and 100 μg/mL streptomycin at 37 °C in a humidified atmosphere of 5% CO_2_. Cells were seeded in 96-well plates at 1 × 10^4^ cells/well for 24 h to allow attachment. The culture medium was removed and replaced by fresh culture medium containing different concentration of the test compounds. After 24 h incubation, 10 μL of MTT solution (5 mg/mL) (ACROS Organics) was added to each well followed by incubation at 37 °C for 4 h. Next, 100 μL of SDS-HCl solution was added to stop the reduction reaction and dissolve the formazan. The absorbance of each well at 595 nm was measured by a DTX880 Multimode Detector. The percentage of cell viability, in relation to untreated cells, was calculated from the absorption data. The MTT assay was performed in triplet (*n* = 3) and the average value of the three measurements was taken.

## Figures and Tables

**Table 1:** Critical Aggregation Concentration (CAC) values for dehydrotripeptides **11**–**18**.

Hydrogelator	Critical Aggregation Concentration (µM)
Suc-D-Hph-L-Phe-*Z*-ΔPhe-OMe, **11**	34.36
Suc-L-Hph-L-Phe-*Z*-ΔPhe-OMe, **12**	40.74
Suc-L-Phe-D-Hph-*Z*-ΔPhe-OMe, **13**	38.19
Suc-L-Phe-L-Hph-*Z*-ΔPhe-OMe, **14**	35.80
Suc-D-Hph-L-Phe-*Z*-ΔPhe-OH, **15**	34.83
Suc-L-Hph-L-Phe-*Z*-ΔPhe-OH, **16**	38.64
Suc-L-Phe-D-Hph-*Z*-ΔPhe-OH, **17**	33.27
Suc-L-Phe-L-Hph-*Z*-ΔPhe-OH, **18**	37.76

**Table 2:** G′ for hydrogels at different concentrations, before (1st sweep) and after breaking (2nd sweep) and recovery percentage from initial G′ value.

Hydrogel	Gelation Concentration wt%	Before Breaking G′ (Pa)	After Breaking G′ (Pa)	Recovery (%)
Suc-*D*-Hph-*L*-Phe-ΔPhe-OMe, **11**	0.2	6.15 × 10^2^	2.15 × 10^2^	35%
	0.6	3.19 × 10^5^	2.88 × 10^4^	9%
Suc-*L*-Hph-*L*-Phe-ΔPhe-OMe, **12**	0.2	4.66 × 10^4^	2.28 × 10^4^	49%
	0.6	5.79 × 10^3^	6.92 × 10^3^	120%
Suc-*L*-Phe-*D*-Hph-ΔPhe-OMe, **13**	0.2	3.62 × 10^4^	1.98 × 10^3^	5%
	0.6	3.15 × 10^5^	3.80 × 10^3^	1%
Suc-*L*-Phe-*L*-Hph-ΔPhe-OMe, **14**	0.2	3.31 × 10^4^	3.82 × 10^5^	1154%
	0.6	2.73 × 10^5^	1.25 × 10^5^	46%
Suc-*D*-Hph-*L*-Phe-ΔPhe-OH, **15**	0.2	3.35 × 10^3^	1.05 × 10^3^	31%
	0.6	3.02 × 10^4^	2.66 × 10^3^	9%
Suc-*L*-Hph-*L*-Phe-ΔPhe-OH, **16**	0.2	1.75 × 10^4^	1.39 × 10^3^	8%
	0.6	1.27 × 10^5^	2.02 × 10^4^	16%
Suc-*L*-Phe-*D*-Hph-ΔPhe-OH, **17**	0.2	1.47 × 10^3^	2.09 × 10^3^	142%
	0.6	3.20 × 10^4^	2.00 × 10^3^	6%
Suc-*L*-Phe-*L*-Hph-ΔPhe-OH, **18**	0.2	2.09 × 10^6^	5.44 × 10^6^	260%
	0.6	8.73 × 10^4^	5.28 × 10^3^	6%

## Data Availability

The original contributions presented in this study are included in the article/Supplementary Material; further inquiries can be directed to the corresponding authors.

## Supplementary Materials

The following supporting information can be downloaded at: https://www.mdpi.com/article/10.3390/gels11030164/s1, Figure S1: Variable temperature 1H-NMR analysis for the α-NH backbone amide signals for peptides; Figure S2: Determination of the critical aggregation concentration (CAC) for dehydrotripeptides **12** (A,B), **13** (C,D), **14** (E,F), **15** (G,H), **16** (I,J), **17** (K,L) and **18** (M,N): A,C,E,G,I,K,M—Steady-state fluorescence spectra (λexc = 280 nm) for dehydropeptide in the concentration range 0.6 to 156 μM; B,D,F,H,J,L,N—semilogarithmic graphical representation of the concentration dependence of the fluorescence intensity ratio (I368/I308); Figure S3: Data for characterisation of hydrogel **12** (0.2wt%) are shown here as illustrative example; Figure S4: Data for characterisation of hydrogel **13** (0.2wt%) are shown here as illustrative example; Figure S5: Data for characterisation of hydrogel **14** (0.2wt%) are shown here as illustrative example; Figure S6: Data for characterisation of hydrogel **15** (0.2wt%) are shown here as illustrative example; Figure S7: Data for characterisation of hydrogel **16** (0.2wt%) are shown here as illustrative example; Figure S8: Data for characterisation of hydrogel **17** (0.2wt%) are shown here as illustrative example; Figure S9: Data for characterisation of hydrogel **18** (0.2wt%) are shown here as illustrative example; Figure S10: Data for characterisation of hydrogel **11** (0.6wt%) are shown here as illustrative example; Figure S11: Data for characterisation of hydrogel **12** (0.6 wt%) are shown here as illustrative example; Figure S12: Data for characterisation of hydrogel **13** (0.6wt%) are shown here as illustrative example; Figure S13: Data for characterisation of hydrogel **14** (0.6wt%) are shown here as illustrative example; Figure S14: Data for characterisation of hydrogel **15** (0.6wt%) are shown here as illustrative example; Figure S15: Data for characterisation of hydrogel **16** (0.6wt%) are shown here as illustrative example; Figure S16: Data for characterisation of hydrogel **17** (0.6wt%) are shown here as illustrative example; Figure S17: Data for characterisation of hydrogel **18** (0.6wt%) are shown here as illustrative example; Table S1: Chemical shift temperature gradient (∆δ/∆T, ppb) values for backbone amides of peptides **15**-**18** in DMSO-d6; Table S2: Chemical shift temperature gradient (∆δ/∆T, ppb) values for backbone amides of peptides **17** and **18** in DMSO-d6 and 10% D2O (v/v); Table S3: Optimized gelation conditions for dehydrotripeptides **11**-**18**; Table S4: Predominant secondary structure elements for hydrogelators **11**-**18** deduced from circular dichroism studies; Synthesis methods.
