# Supplementary material for: Tripeptides Featuring Dehydrophenylalanine and Homophenylalanine: Homo- Versus Hetero-Chirality and Sequence Effects on Self-Assembly and Gelation
Source: Gels. 2025 Feb 24;11(3):164. doi: 10.3390/gels11030164 (PMC11942182; doi:10.3390/gels11030164)
Supplement: Supplementary file 1 [file gels-11-00164-s001.zip › gels-3449300-supplementary.pdf]

Supporting Information

Tripeptides Featuring Dehydrophenylalanine and Homophenylalanine: Homo- Versus Hetero-Chirality and Sequence Effects on Self-Assembly and Gelation

André F. Carvalho [1], Teresa Pereira [1], Carlos Oliveira [1], Pedro Figueiredo [2,3], Alexandra Carvalho [2,4], David M. Pereira [5], Loic Hilliou [6], Manuel Bañobre-López [7], Bing Xu [8], Paula M. T. Ferreira [1,*] and José A. Martins [1,*]

1 Center of Chemistry, University of Minho, 4710-057 Braga, Portugal; carlos.oliveira.6b@gmail.com (C.O.)
2 CNC—Center for Neuroscience and Cell Biology, Institute for Interdisciplinary Research (IIIUC), University of Coimbra, 3004-504 Coimbra, Portugal
3 PhD Programme in Experimental Biology and Biomedicine, Institute for Interdisciplinary Research (IIIUC), University of Coimbra, Casa Costa Alemão, 3030-789 Coimbra, Portugal
4 Almac Sciences, Department of Biocatalysis and Isotope Chemistry, Almac House, 20 Seagoe Industrial Estate, Craigavon BT63 5QD, UK
5 REQUIMTE/LAQV, Laboratório de Farmacognosia, Departamento de Química, Faculdade de Farmácia, Universidade do Porto, R. Jorge Viterbo Ferreira, n 228, 4050-313 Porto, Portugal
6 Institute for Polymers and Composites, University of Minho, 4800-058 Guimarães, Portugal;
loic@dep.uminho.pt
7 International Iberian Nanotechnology Laboratory (INL), Av. Mestre José Veiga s/n, 4715-330 Braga, Portugal; manuel.banobre@inl.int (MB)
8 Department of Chemistry, Brandeis University, 415 South Street, Waltham, MA 02453, USA
* Correspondence: pmf@quimica.uminho.pt (P.M.T.F.); jmartins@quimica.uminho.pt (J.A.M.)

1. Variable-temperature ^{1}H NMR spectroscopy

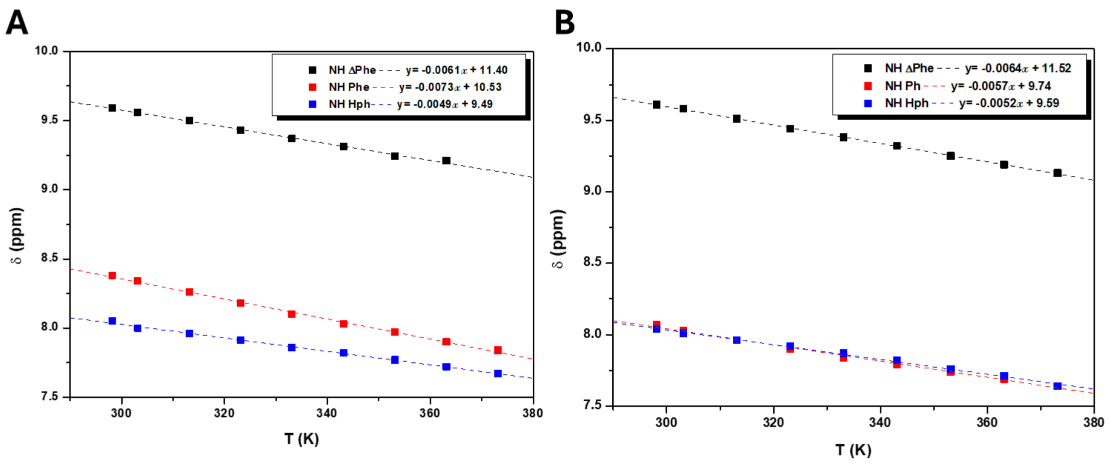

Figure S1. Variable temperature ^{1}H-NMR analysis for the α-NH backbone amide signals for peptides: A) **15**; B) **16** in DMSO-d_6.

Table S1. Chemical shift temperature gradient (Δδ/ΔT, ppb) values for backbone amides of peptides **15**-**18** in DMSO-d_6.

Dehydrotripeptide	Δδ/ΔT (ppb)		
	NH ΔPhe	NH Phe	NH Hph
Suc-*D*-Hph-*L*-Phe-ΔPhe-OH, **15**	-6.1	-7.3	-4.9
Suc-*L*-Hph-*L*-Phe-ΔPhe-OH, **16**	-6.4	-5.7	-5.2
Suc-*L*-Phe-*D*-Hph-ΔPhe-OH, **17**	-6.5	-5.1	-7.0
Suc-*L*-Phe-*L*-Hph-ΔPhe-OH, **18**	-6.4	-4.9	-6.3

Table S2. Chemical shift temperature gradient (Δδ/ΔT, ppb) values for backbone amides of peptides **17** and **18** in DMSO-d_6 and 10% D_2O (v/v).

Dehydrotripeptide	Δδ/ΔT (ppb)		
	NH ΔPhe	NH Phe	NH Hph
Suc-*L*-Phe-*D*-Hph-ΔPhe-OH, **17**	-6.0	-6.0	-5.0
Suc-*L*-Phe-*L*-Hph-ΔPhe-OH, **18**	-6.6	-6.3	-5.5

2. Hydrogelation

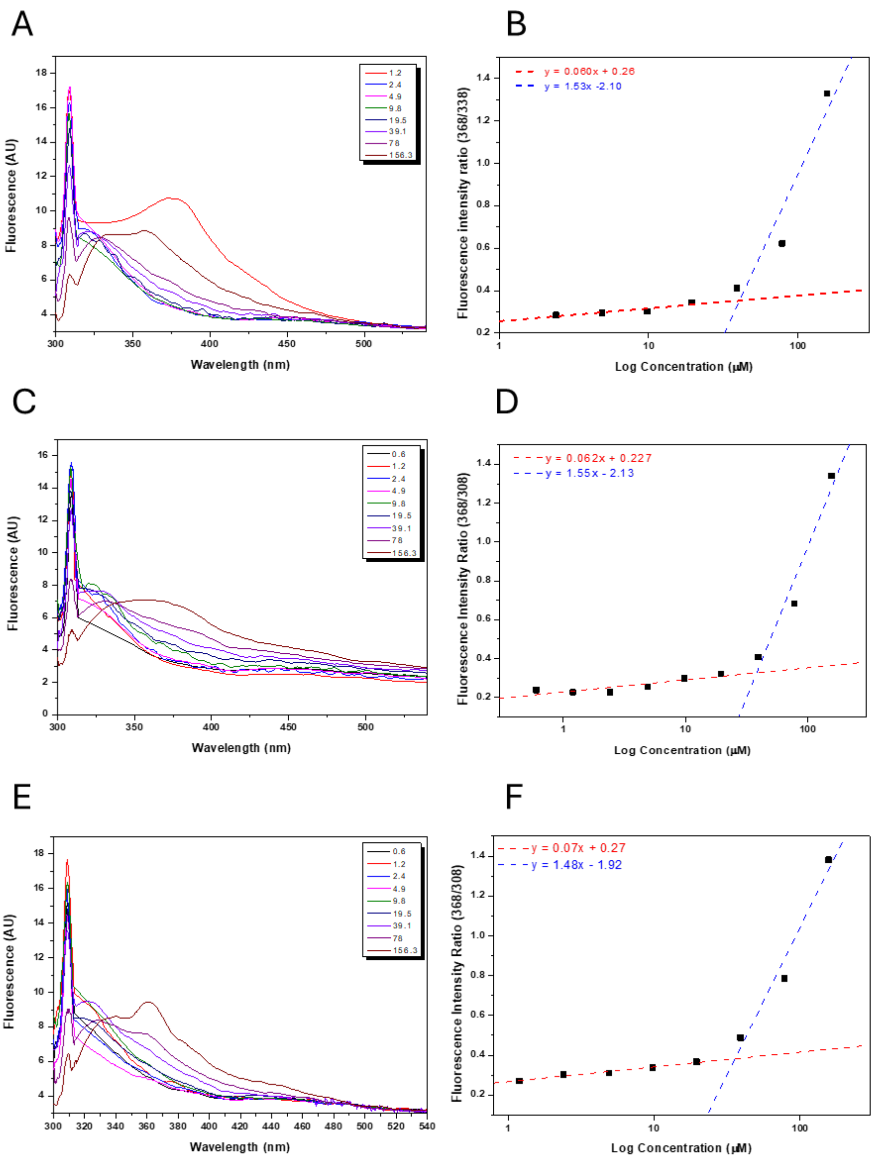

A
B
C
D
E
F
Fluorescence (AU)
Wavelength (nm)
0.6
1.2
2.4
4.9
9.8
19.5
39.1
78
156.3
Fluorescence intensity ratio (368/338)
y = 0.060x + 0.26
y = 1.53x -2.10
Fluorescence Intensity Ratio (368/308)
y = 0.062x + 0.227
y = 1.55x - 2.13
y = 0.07x + 0.27
y = 1.48x - 1.92
Log Concentration (µM)

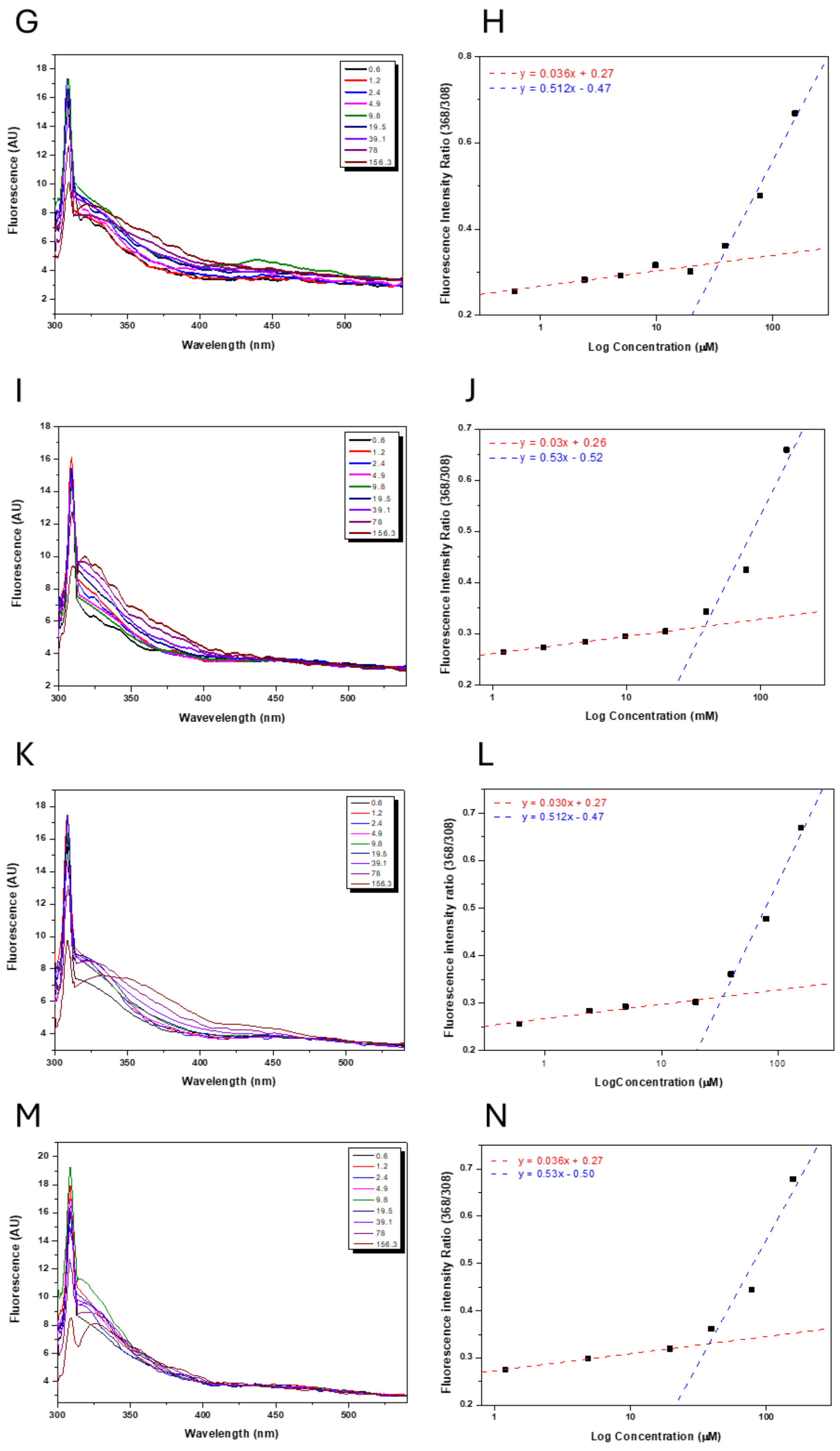

Figure S2. Determination of the critical aggregation concentration (CAC) for dehydrotripeptides **12** (A,B), **13** (C,D) , **14** (E,F), **15** (G,H), **16** (I,J), **17** (K,L) and **18** (M,N): A,C,E,G,I,K,M - Steady-state fluorescence spectra (λ_{exc} = 280 nm) for dehydropeptide in the concentration range 0.6 to 156 μM; B,D,F,H,J,L,N – semilogarithmic graphical representation of the concentration dependence of the fluorescence intensity ratio (I_{368}/I_{308}).

Table S3. Optimized gelation conditions for dehydrotripeptides **11**-**18**.

Hydrogelator	CGC / wt% (mM)	[GdL] / wt%	pH	cLogP*
Suc-*D*-Hph-*L*-Phe-*Z*-ΔPhe-OMe, **11**	0.2 (3.4 mM)	0.2	5.4	3.84
Suc-*L*-Hph-*L*-Phe-*Z*-ΔPhe-OMe, **12**	0.2 (3.4 mM)	0.2	4.5	3.84
Suc-*L*-Phe-*D*-Hph-*Z*-ΔPhe-OMe, **13**	0.2 (3.4 mM)	0.2	5.5	3.84
Suc-*L*-Phe-*L*-Hph-*Z*-ΔPhe-OMe, **14**	0.2 (3.4 mM)	0.2	4.8	3.84
Suc-*D*-Hph-*L*-Phe-*Z*-ΔPhe-OH, **15**	0.2 (3.5 mM)	0.3	4.7	3.22
Suc-*L*-Hph-*L*-Phe-*Z*-ΔPhe-OH, **16**	0.2 (3.5 mM)	0.3	4.7	3.22
Suc-*L*-Phe-*D*-Hph-*Z*-ΔPhe-OH, **17**	0.2 (3.5 mM)	0.3	4.7	3.22
Suc-*L*-Phe-*L*-Hph-*Z*-ΔPhe-OH, **18**	0.2 (3.5 mM)	0.3	5.0	3.22

*cLogP value obtained from https//molinspiration.com

3. Circular Dichroism

Table S4. Predominant secondary structure elements for hydrogelators **11**-**18** deduced from circular dichroism studies

Hydrogelator	Predominant Secondary Structure
Suc-*D*-Hph-*L*-Phe-Z-ΔPhe-OMe, **11**	Random Coil
Suc-*L*-Hph-*L*-Phe-*Z*-ΔPhe-OMe, **12**	α-helix and random coil
Suc-*L*-Phe-*D*-Hph-Z-ΔPhe-OMe, **13**	β-sheet
Suc-*L*-Phe-*L*-Hph-*Z*-ΔPhe-OMe, **14**	α-helix and random coil
Suc-*D*-Hph-*L*-Phe-Z-ΔPhe-OH, **15**	Random Coil
Suc-*L*-Hph-*L*-Phe-Z-ΔPhe-OH, **16**	α-helix and random coil
Suc-*L*-Phe-*D*-Hph-Z-ΔPhe-OH, **17**	β-sheet
Suc-*L*-Phe-*L*-Hph-Z-ΔPhe-OH, **18**	α-helix and random coil

4. Rheology

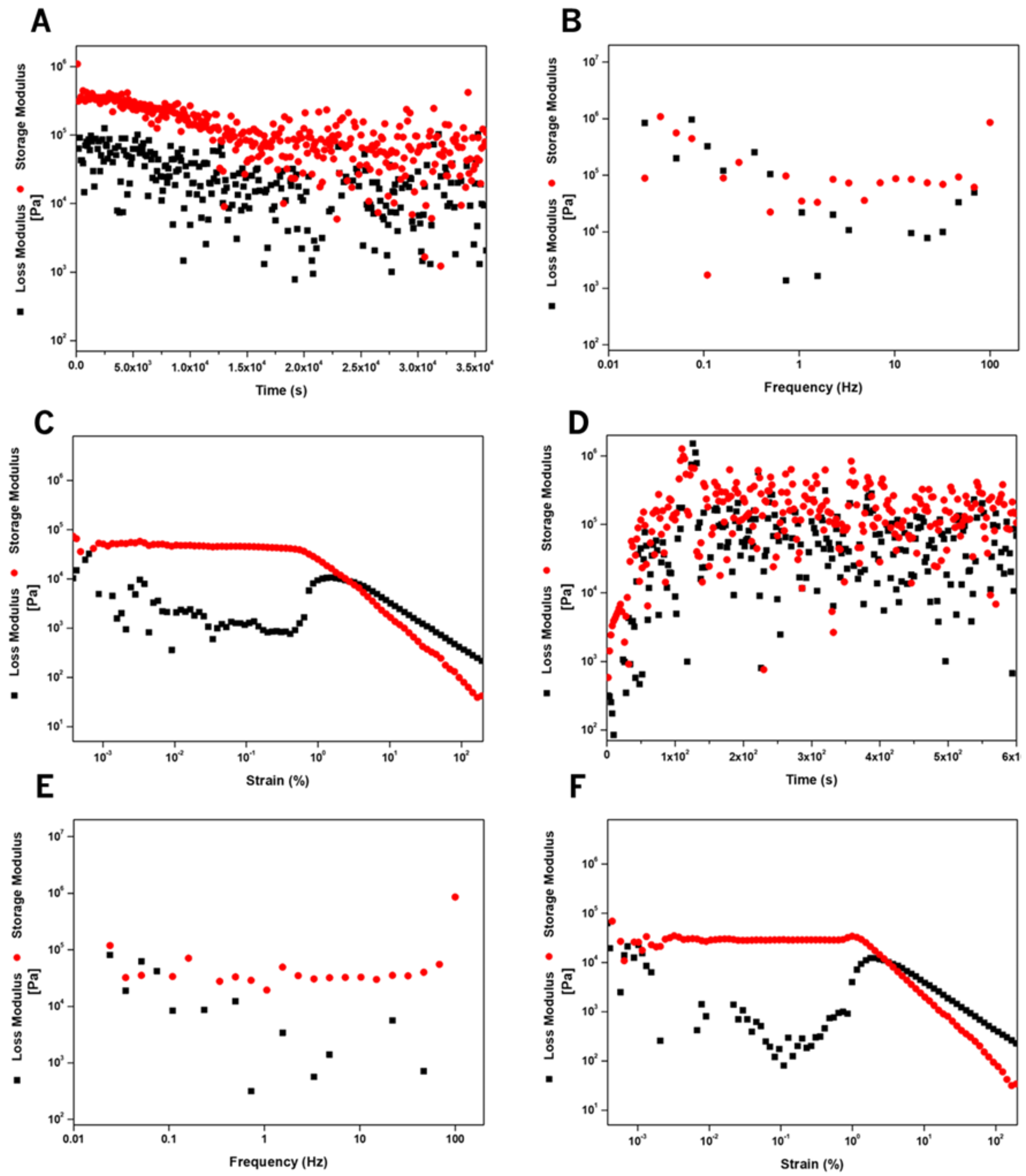

Figure S3. Data for characterisation of hydrogel **12** (0.2wt%) : A- Kinetic study at constant frequency (f =1 Hz) and strain (1x10^{-4} %); B - Mechanical frequency sweep, at constant strain (1x10^{-4} %); C- Large amplitude oscillatory shear(Strain sweep) at constant frequency (f =1 Hz); D- Hydrogel kinetics reformation study at constant frequency (f =1 Hz) and strain (1x10^{-4} %); E- Mechanical frequency sweep of reformed hydrogel , at constant strain (1x10^{-3} %); F- Large amplitude oscillatory shear(Strain sweep) for reformed hydrogel at constant frequency (f =1 Hz).

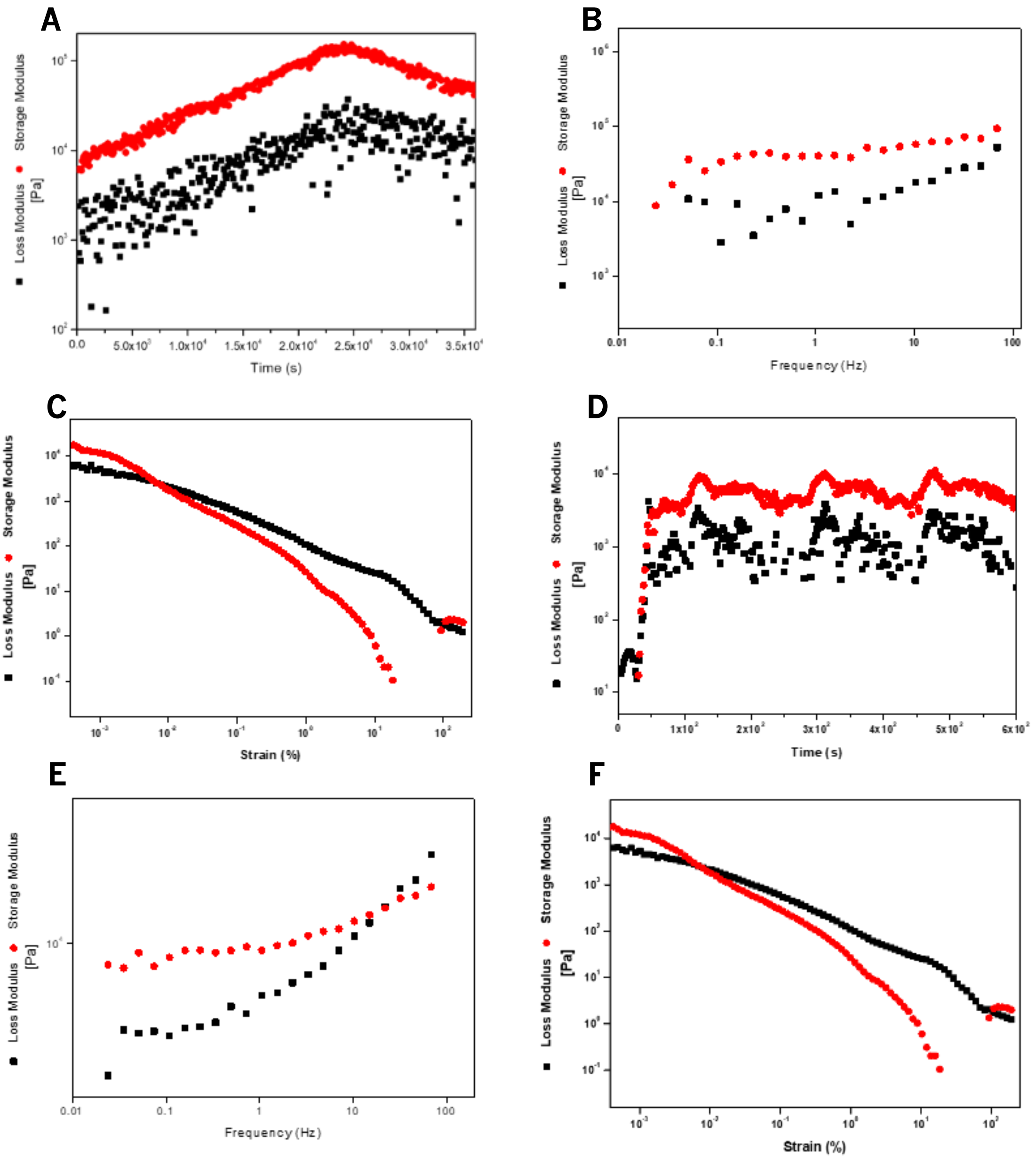

Figure S4. Data for characterisation of hydrogel **13** (0.2wt%) : A- Kinetic study at constant frequency (f =1 Hz) and strain (1x10^{-4} %); B - Mechanical frequency sweep, at constant strain (1x10^{-4} %); C- Large amplitude oscillatory shear(Strain sweep) at constant frequency (f =1 Hz); D- Hydrogel kinetics reformation study at constant frequency (f =1 Hz) and strain (1x10^{-4} %); E- Mechanical frequency sweep of reformed hydrogel , at constant strain (1x10^{-3} %); F- Large amplitude oscillatory shear(Strain sweep) for reformed hydrogel at constant frequency (f =1 Hz).

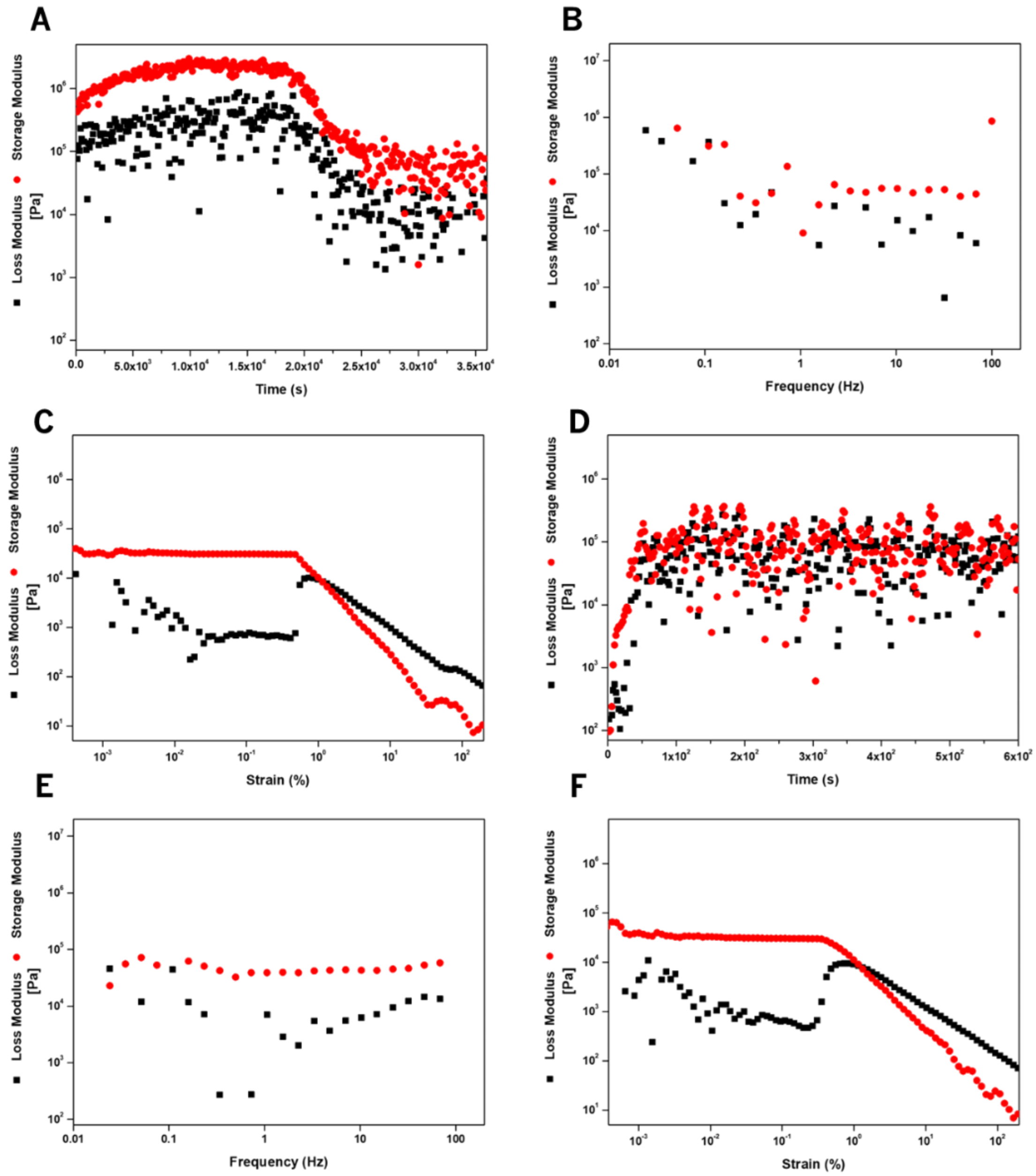

Figure S5. Data for characterisation of hydrogel **14** (0.2wt%) : A- Kinetic study at constant frequency (f =1 Hz) and strain (1x10^{-4} %); B - Mechanical frequency sweep, at constant strain (1x10^{-4} %); C- Large amplitude oscillatory shear(Strain sweep) at constant frequency (f =1 Hz); D- Hydrogel kinetics reformation study at constant frequency (f =1 Hz) and strain (1x10^{-4} %); E- Mechanical frequency sweep of reformed hydrogel , at constant strain (1x10^{-3} %); F- Large amplitude oscillatory shear(Strain sweep) for reformed hydrogel at constant frequency (f =1 Hz).

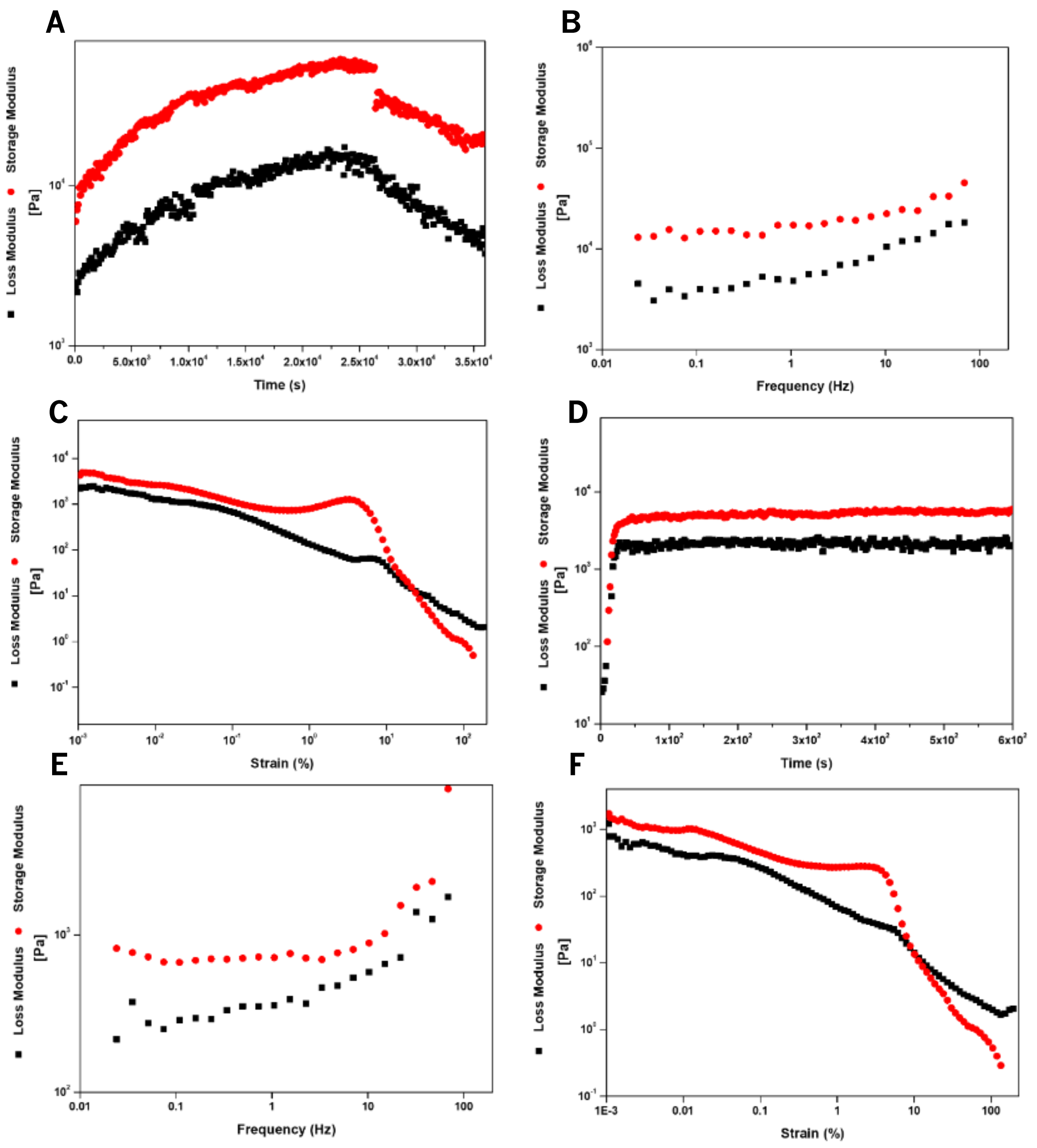

Figure S6. Data for characterisation of hydrogel **15** (0.2wt%) : A- Kinetic study at constant frequency (f =1 Hz) and strain (1x10^{-4} %); B - Mechanical frequency sweep, at constant strain (1x10^{-4} %); C- Large amplitude oscillatory shear(Strain sweep) at constant frequency (f =1 Hz); D- Hydrogel kinetics reformation study at constant frequency (f =1 Hz) and strain (1x10^{-4} %); E- Mechanical frequency sweep of reformed hydrogel , at constant strain (1x10^{-3} %); F- Large amplitude oscillatory shear(Strain sweep) for reformed hydrogel at constant frequency (f =1 Hz).

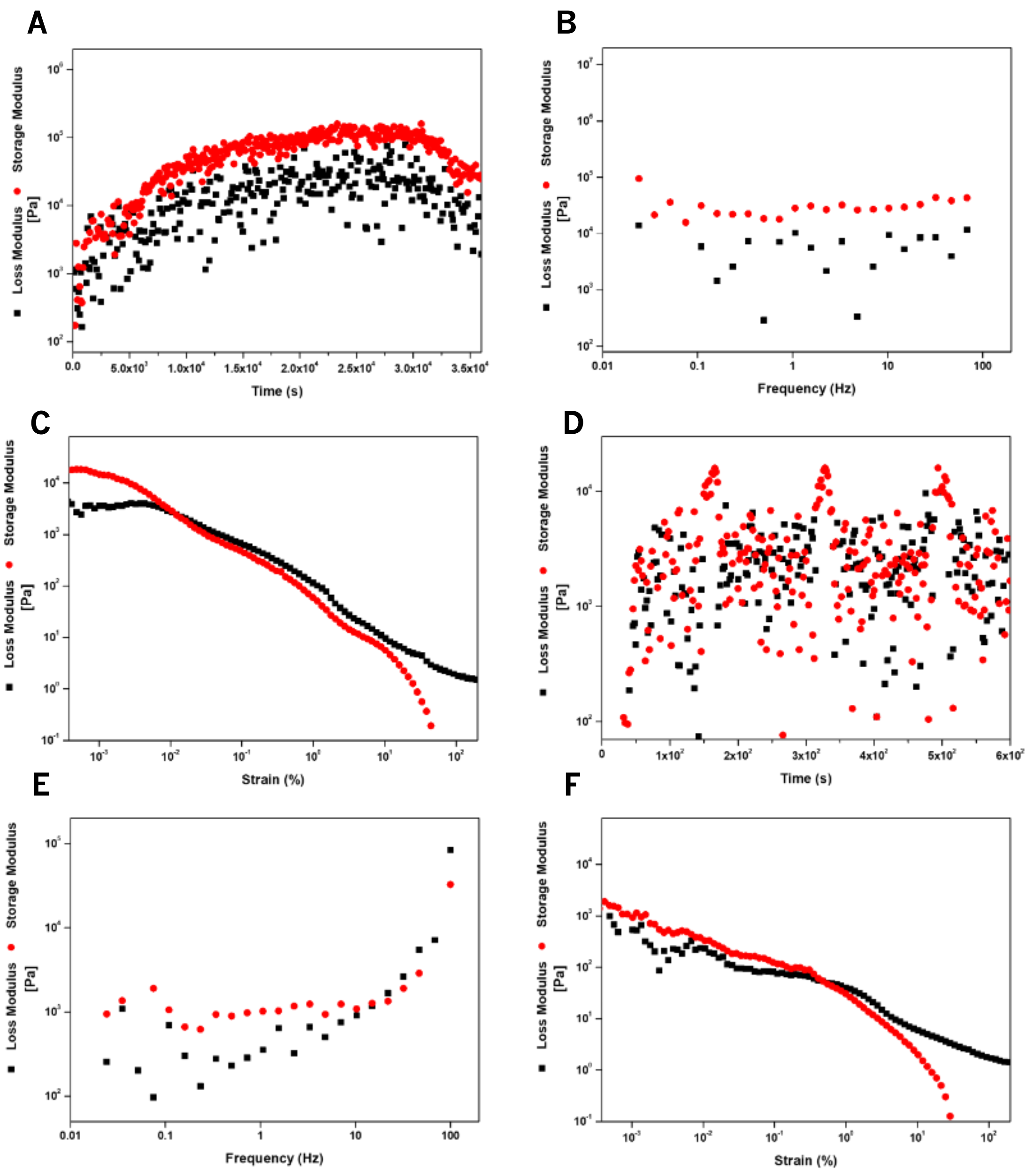

Figure S7. Data for characterisation of hydrogel **16** (0.2wt%) : A- Kinetic study at constant frequency (f =1 Hz) and strain ($1x10^{-4}$ %); B - Mechanical frequency sweep, at constant strain ($1x10^{-4}$ %); C- Large amplitude oscillatory shear(Strain sweep) at constant frequency (f =1 Hz); D- Hydrogel kinetics reformation study at constant frequency (f =1 Hz) and strain ($1x10^{-4}$ %); E- Mechanical frequency sweep of reformed hydrogel , at constant strain ($1x10^{-3}$ %); F- Large amplitude oscillatory shear(Strain sweep) for reformed hydrogel at constant frequency (f =1 Hz).

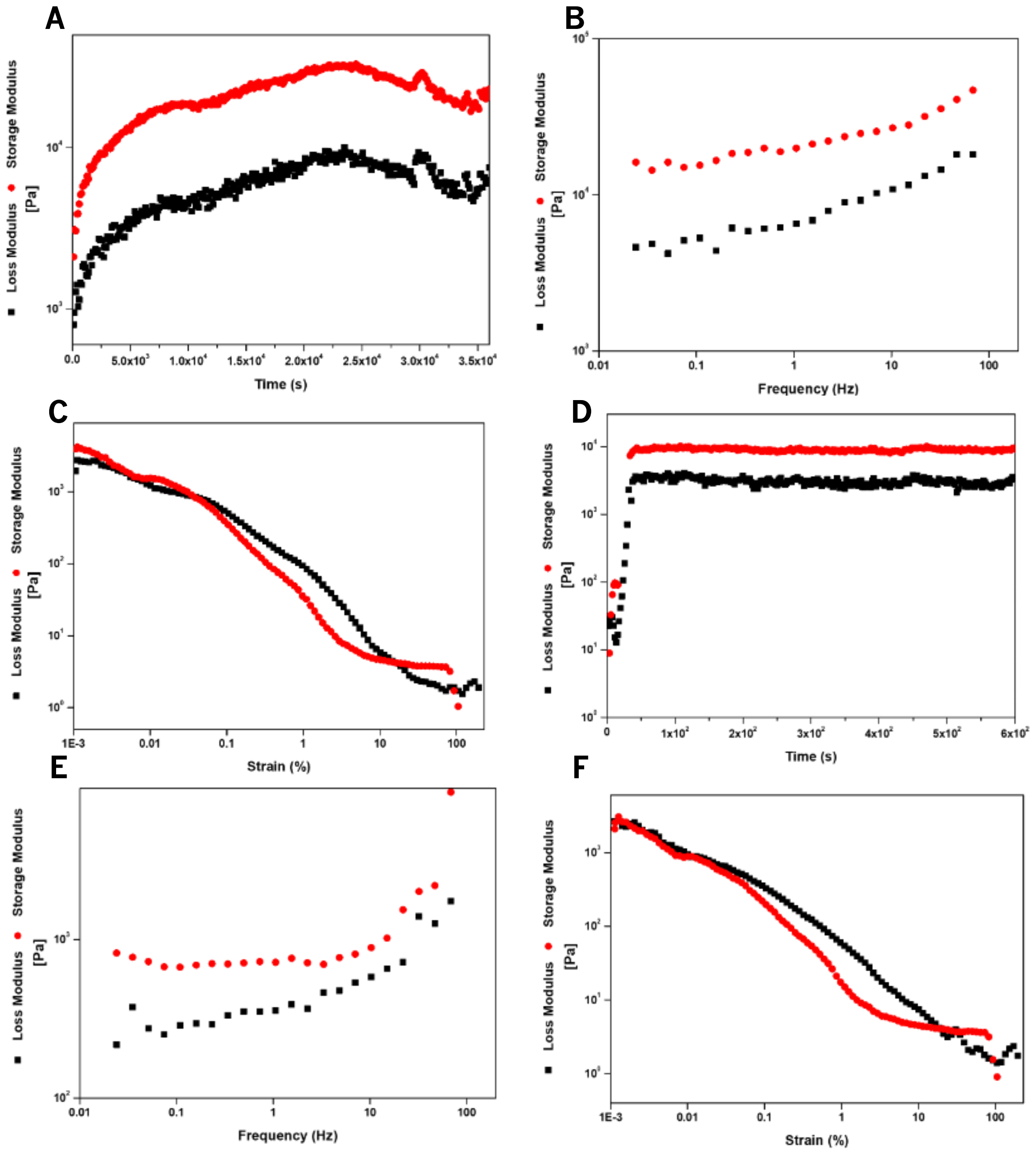

Figure S8. Data for characterisation of hydrogel **17** (0.2wt%) : A- Kinetic study at constant frequency (f =1 Hz) and strain ($1x10^{-4}$ %); B - Mechanical frequency sweep, at constant strain ($1x10^{-4}$ %); C- Large amplitude oscillatory shear(Strain sweep) at constant frequency (f =1 Hz); D- Hydrogel kinetics reformation study at constant frequency (f =1 Hz) and strain ($1x10^{-4}$ %); E- Mechanical frequency sweep of reformed hydrogel , at constant strain ($1x10^{-3}$ %); F- Large amplitude oscillatory shear(Strain sweep) for reformed hydrogel at constant frequency (f =1 Hz).

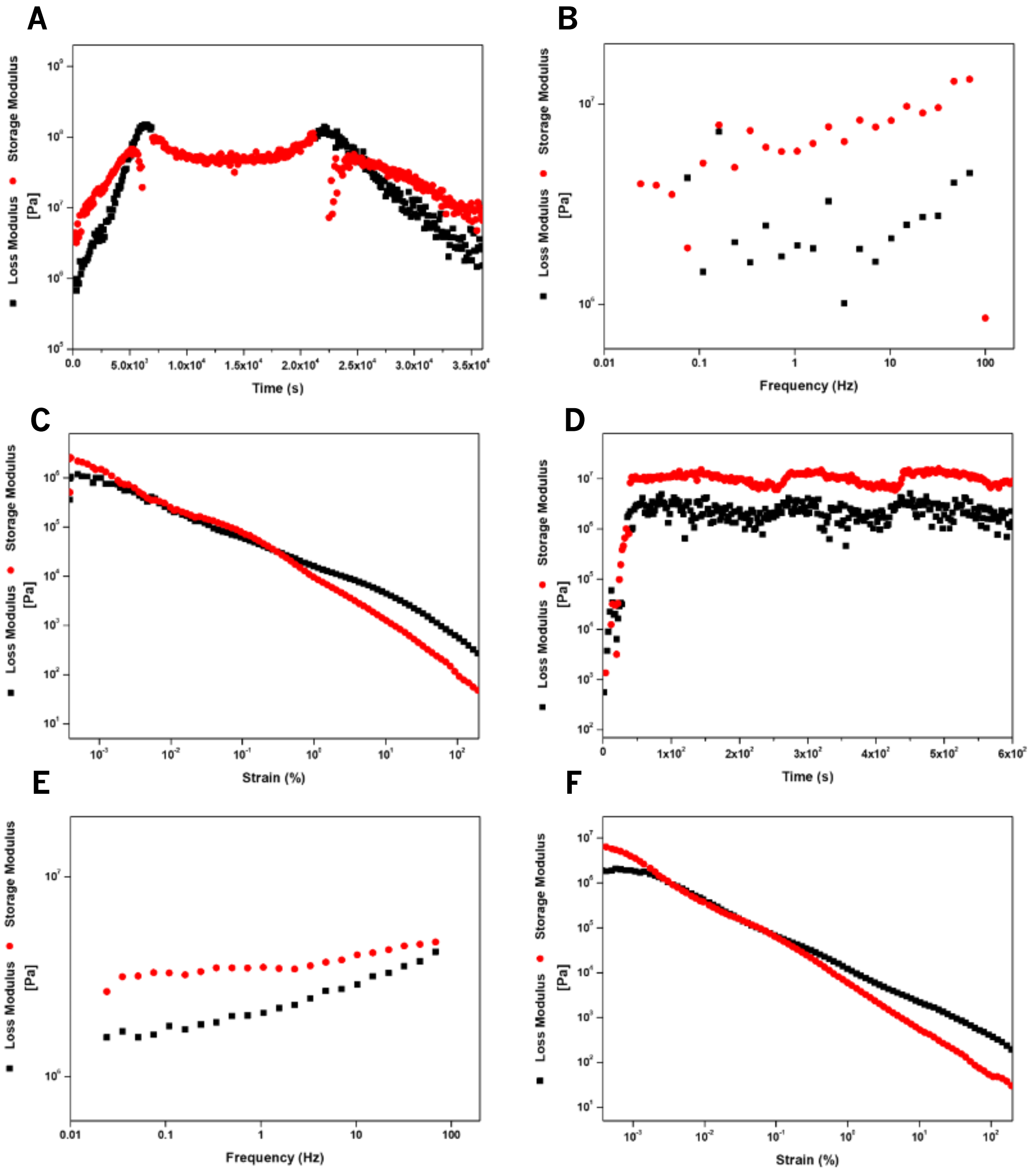

Figure S9. Data for characterisation of hydrogel **18** (0.2wt%) : A- Kinetic study at constant frequency (f =1 Hz) and strain (1x10^{-4} %); B - Mechanical frequency sweep, at constant strain (1x10^{-4} %); C- Large amplitude oscillatory shear(Strain sweep) at constant frequency (f =1 Hz); D- Hydrogel kinetics reformation study at constant frequency (f =1 Hz) and strain (1x10^{-4} %); E- Mechanical frequency sweep of reformed hydrogel , at constant strain (1x10^{-3} %); F- Large amplitude oscillatory shear(Strain sweep) for reformed hydrogel at constant frequency (f =1 Hz).

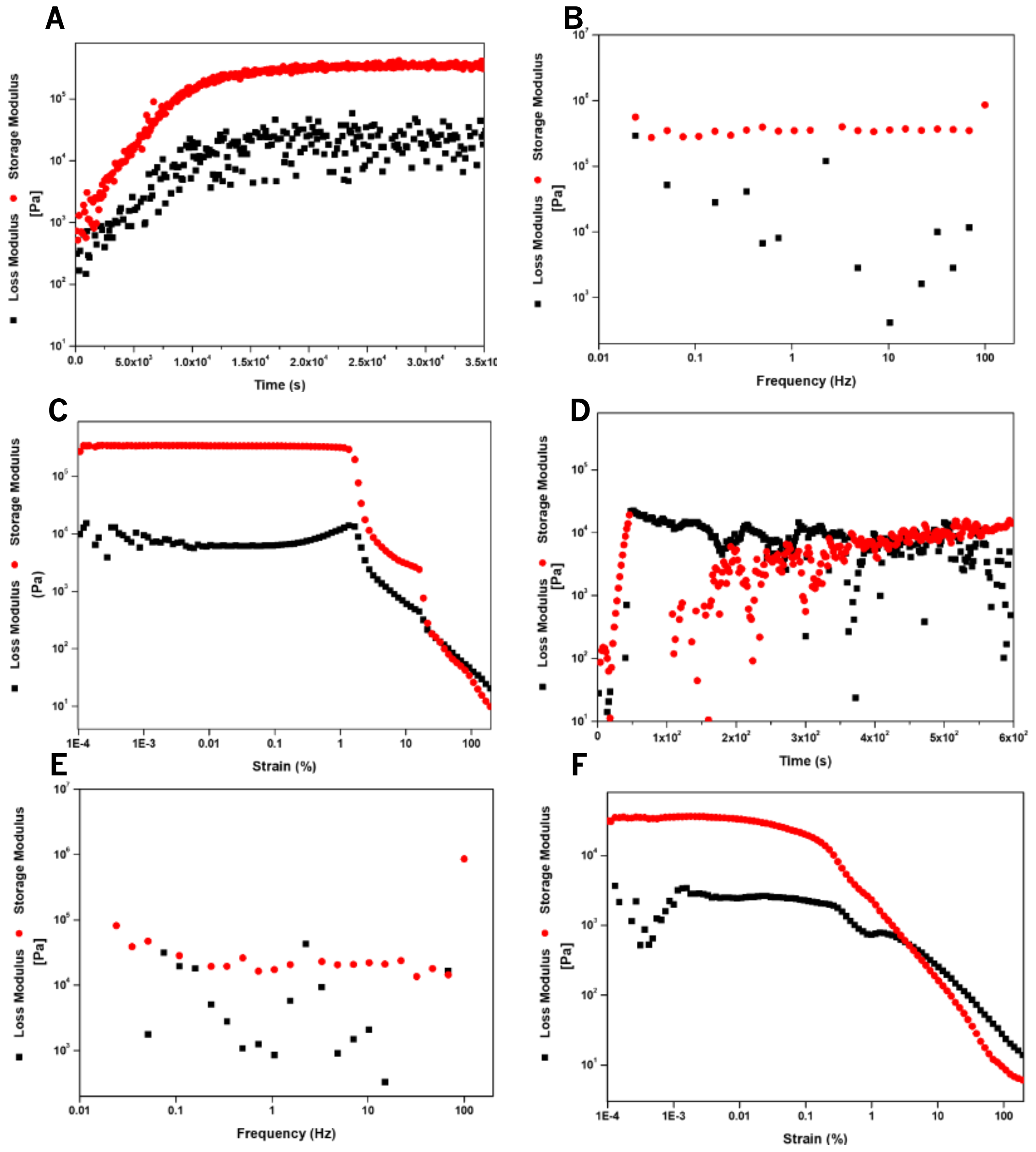

Figure S10. Data for characterisation of hydrogel **11** (0.6wt%) : A- Kinetic study at constant frequency (f =1 Hz) and strain (1x10^{-4} %); B - Mechanical frequency sweep, at constant strain (1x10^{-4} %); C- Large amplitude oscillatory shear(Strain sweep) at constant frequency (f =1 Hz); D- Hydrogel kinetics reformation study at constant frequency (f =1 Hz) and strain (1x10^{-4} %); E- Mechanical frequency sweep of reformed hydrogel , at constant strain (1x10^{-3} %); F- Large amplitude oscillatory shear(Strain sweep) for reformed hydrogel at constant frequency (f =1 Hz).

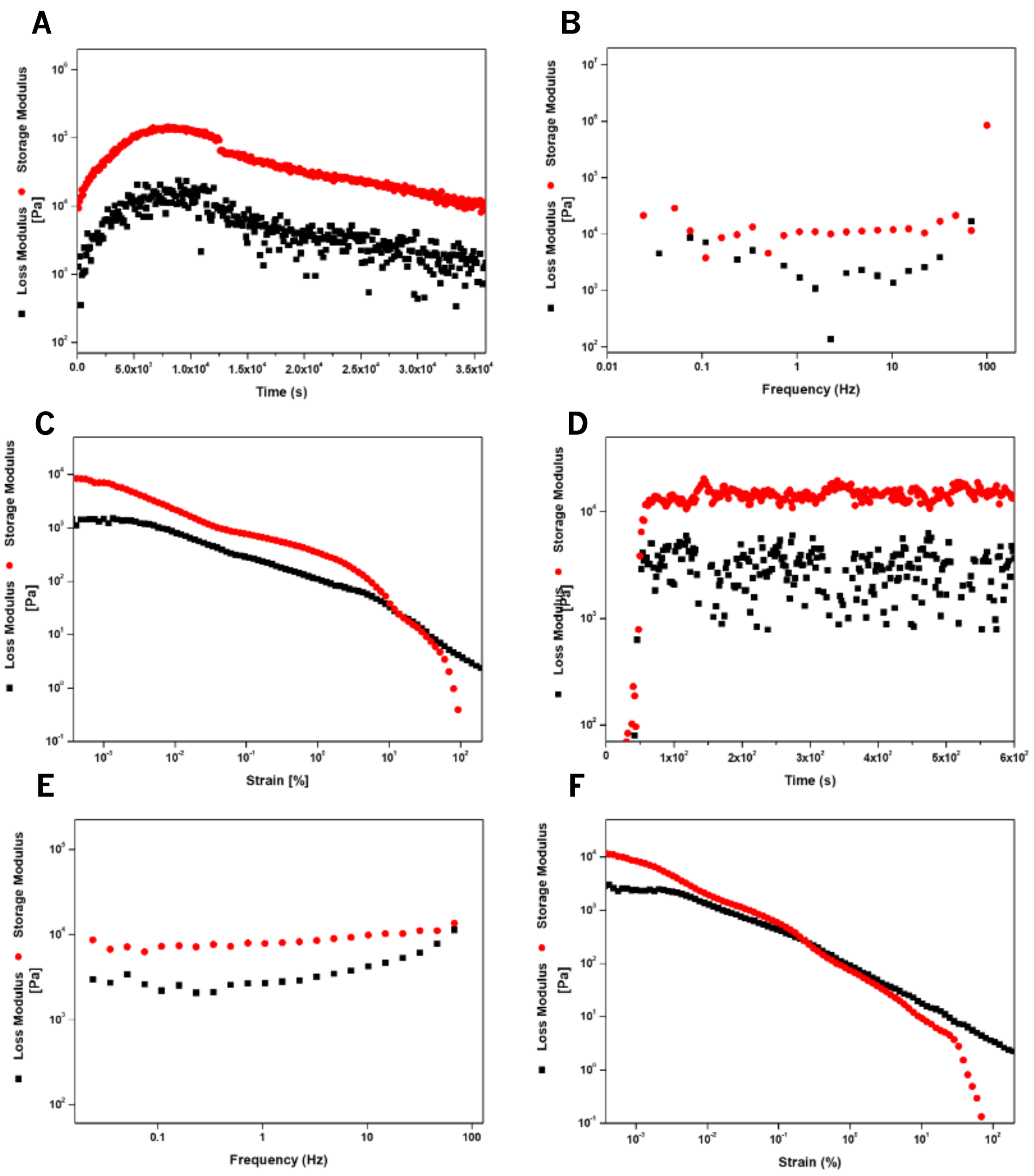

Figure S11. Data for characterisation of hydrogel **12** (0.6 wt%) : A- Kinetic study at constant frequency (f =1 Hz) and strain (1x10^{-4} %); B - Mechanical frequency sweep, at constant strain (1x10^{-4} %); C- Large amplitude oscillatory shear(Strain sweep) at constant frequency (f =1 Hz); D- Hydrogel kinetics reformation study at constant frequency (f =1 Hz) and strain (1x10^{-4} %); E- Mechanical frequency sweep of reformed hydrogel , at constant strain (1x10^{-3} %); F- Large amplitude oscillatory shear(Strain sweep) for reformed hydrogel at constant frequency (f =1 Hz).

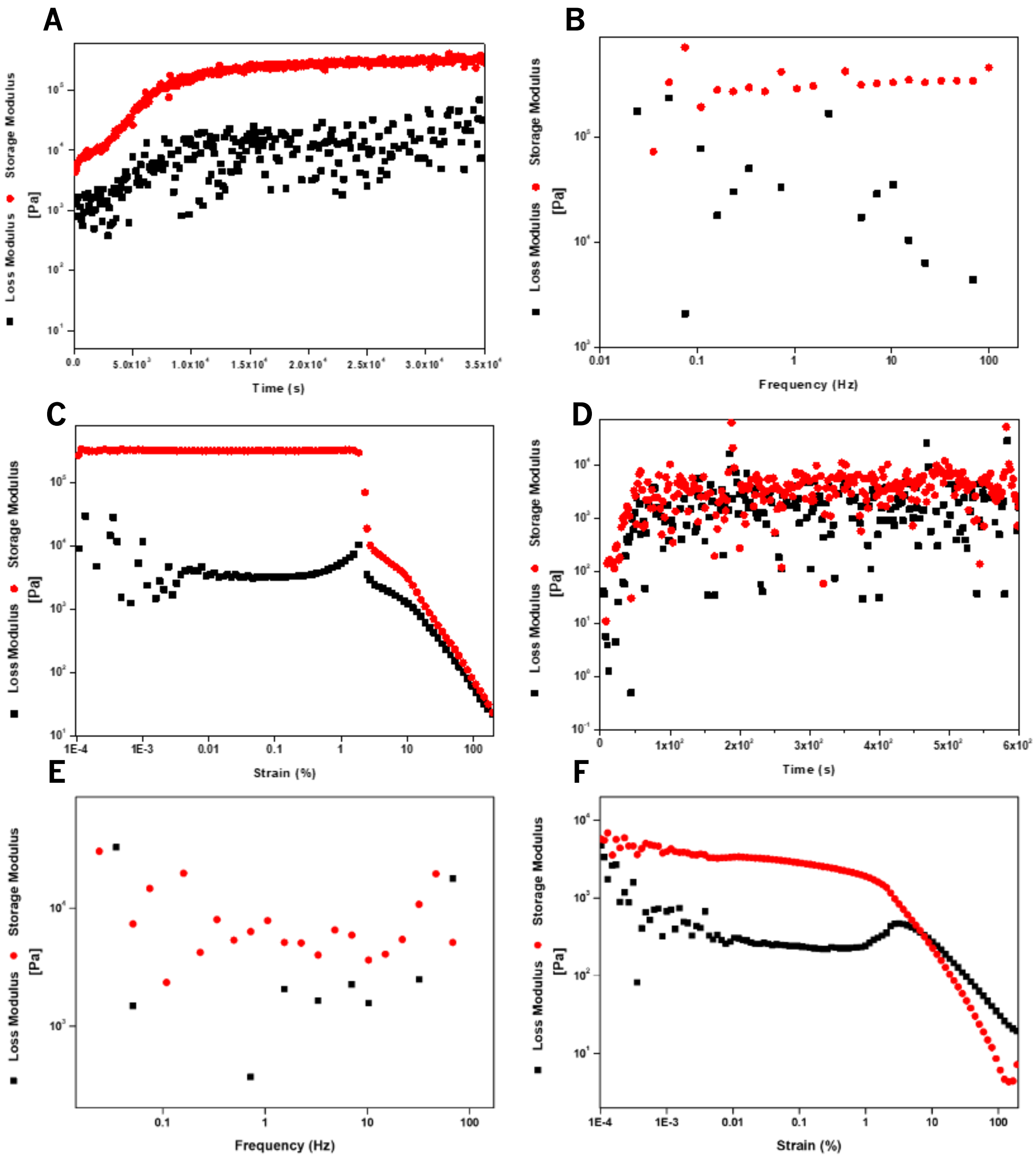

Figure S12. Data for characterisation of hydrogel **13** (0.6wt%) : A- Kinetic study at constant frequency (f =1 Hz) and strain ($1x10^{-4}$ %); B - Mechanical frequency sweep, at constant strain ($1x10^{-4}$ %); C- Large amplitude oscillatory shear(Strain sweep) at constant frequency (f =1 Hz); D- Hydrogel kinetics reformation study at constant frequency (f =1 Hz) and strain ($1x10^{-4}$ %); E- Mechanical frequency sweep of reformed hydrogel , at constant strain ($1x10^{-3}$ %); F- Large amplitude oscillatory shear(Strain sweep) for reformed hydrogel at constant frequency (f =1 Hz).

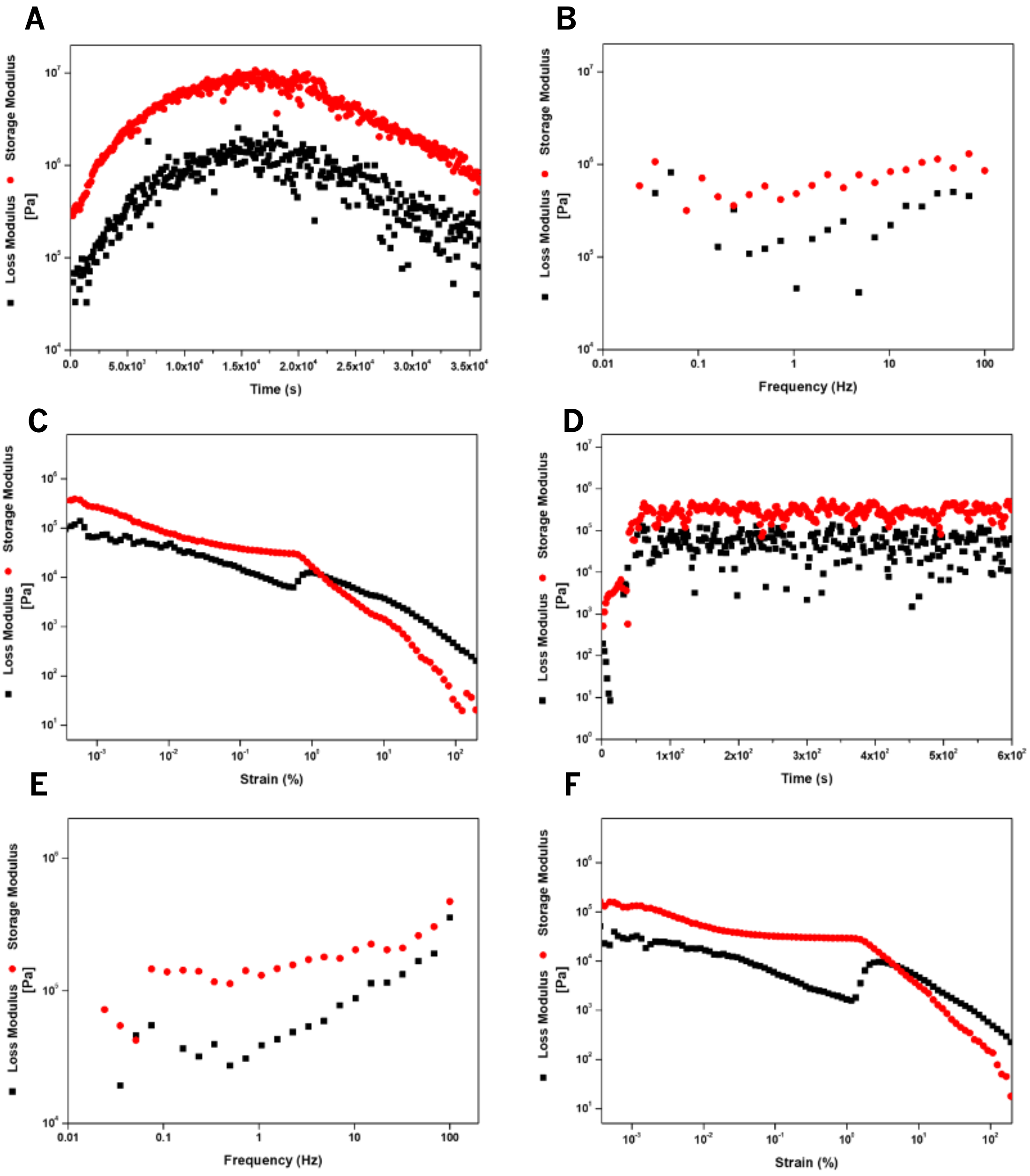

Figure S13. Data for characterisation of hydrogel 14 (0.6wt%) : A- Kinetic study at constant frequency (f =1 Hz) and strain (1x10^{-4} %); B - Mechanical frequency sweep, at constant strain (1x10^{-4} %); C- Large amplitude oscillatory shear(Strain sweep) at constant frequency (f =1 Hz); D- Hydrogel kinetics reformation study at constant frequency (f =1 Hz) and strain (1x10^{-4} %); E- Mechanical frequency sweep of reformed hydrogel , at constant strain (1x10^{-3} %); F- Large amplitude oscillatory shear(Strain sweep) for reformed hydrogel at constant frequency (f =1 Hz).

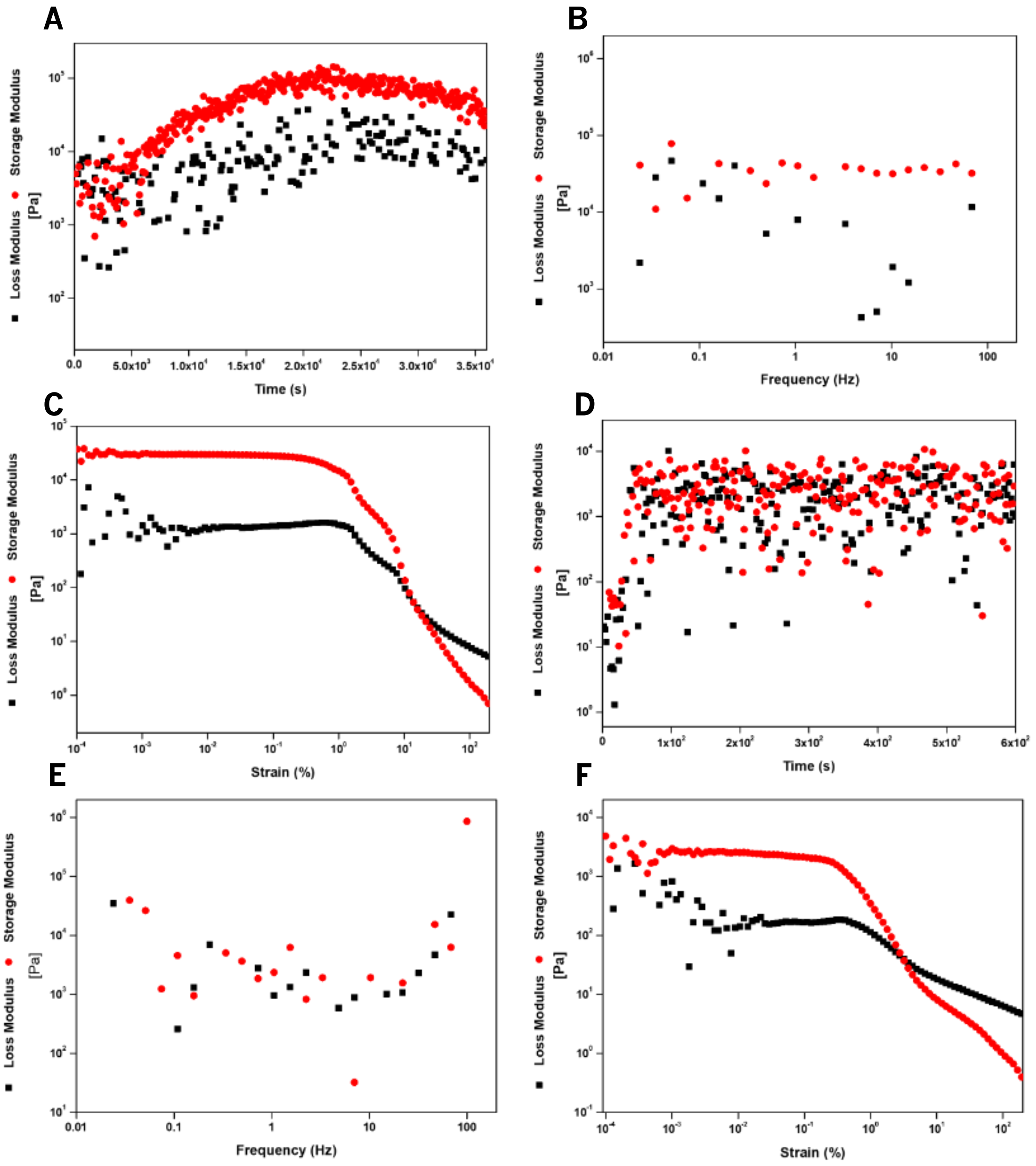

Figure S14. Data for characterisation of hydrogel **15** (0.6wt%) : A- Kinetic study at constant frequency (f =1 Hz) and strain ($1x10^{-4}$ %); B - Mechanical frequency sweep, at constant strain ($1x10^{-4}$ %); C- Large amplitude oscillatory shear(Strain sweep) at constant frequency (f =1 Hz); D- Hydrogel kinetics reformation study at constant frequency (f =1 Hz) and strain ($1x10^{-4}$ %); E- Mechanical frequency sweep of reformed hydrogel , at constant strain ($1x10^{-3}$ %); F- Large amplitude oscillatory shear(Strain sweep) for reformed hydrogel at constant frequency (f =1 Hz).

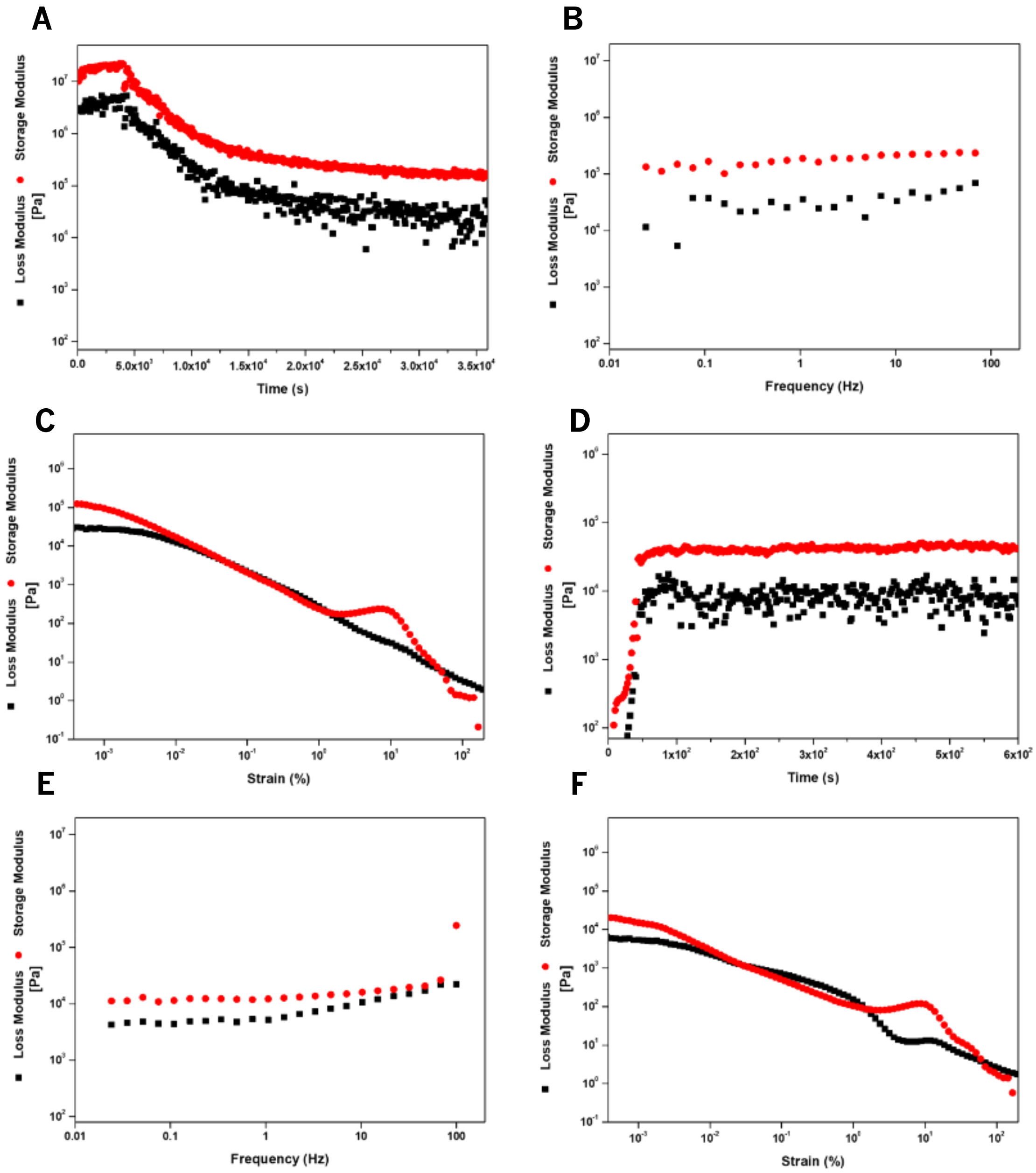

Figure S15. Data for characterisation of hydrogel **16** (0.6wt%) : A- Kinetic study at constant frequency (f =1 Hz) and strain ($1x10^{-4}$ %); B - Mechanical frequency sweep, at constant strain ($1x10^{-4}$ %); C- Large amplitude oscillatory shear(Strain sweep) at constant frequency (f =1 Hz); D- Hydrogel kinetics reformation study at constant frequency (f =1 Hz) and strain ($1x10^{-4}$ %); E- Mechanical frequency sweep of reformed hydrogel , at constant strain ($1x10^{-3}$ %); F- Large amplitude oscillatory shear(Strain sweep) for reformed hydrogel at constant frequency (f =1 Hz).

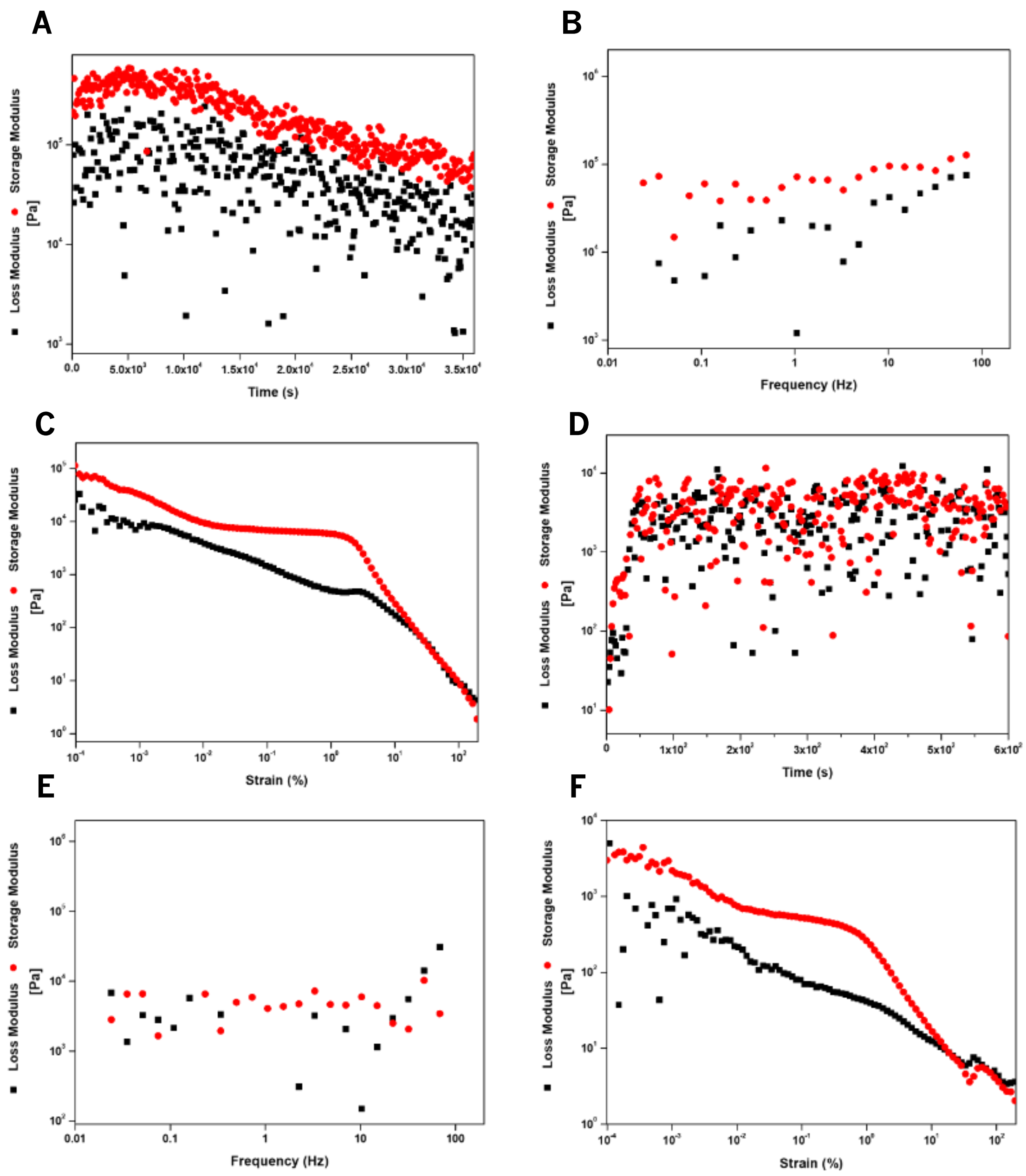

Figure S16. Data for characterisation of hydrogel **17** (0.6wt%) : A- Kinetic study at constant frequency (f =1 Hz) and strain (1x10^{-4} %); B - Mechanical frequency sweep, at constant strain (1x10^{-4} %); C- Large amplitude oscillatory shear(Strain sweep) at constant frequency (f =1 Hz); D- Hydrogel kinetics reformation study at constant frequency (f =1 Hz) and strain (1x10^{-4} %); E- Mechanical frequency sweep of reformed hydrogel , at constant strain (1x10^{-3} %); F- Large amplitude oscillatory shear(Strain sweep) for reformed hydrogel at constant frequency (f =1 Hz).

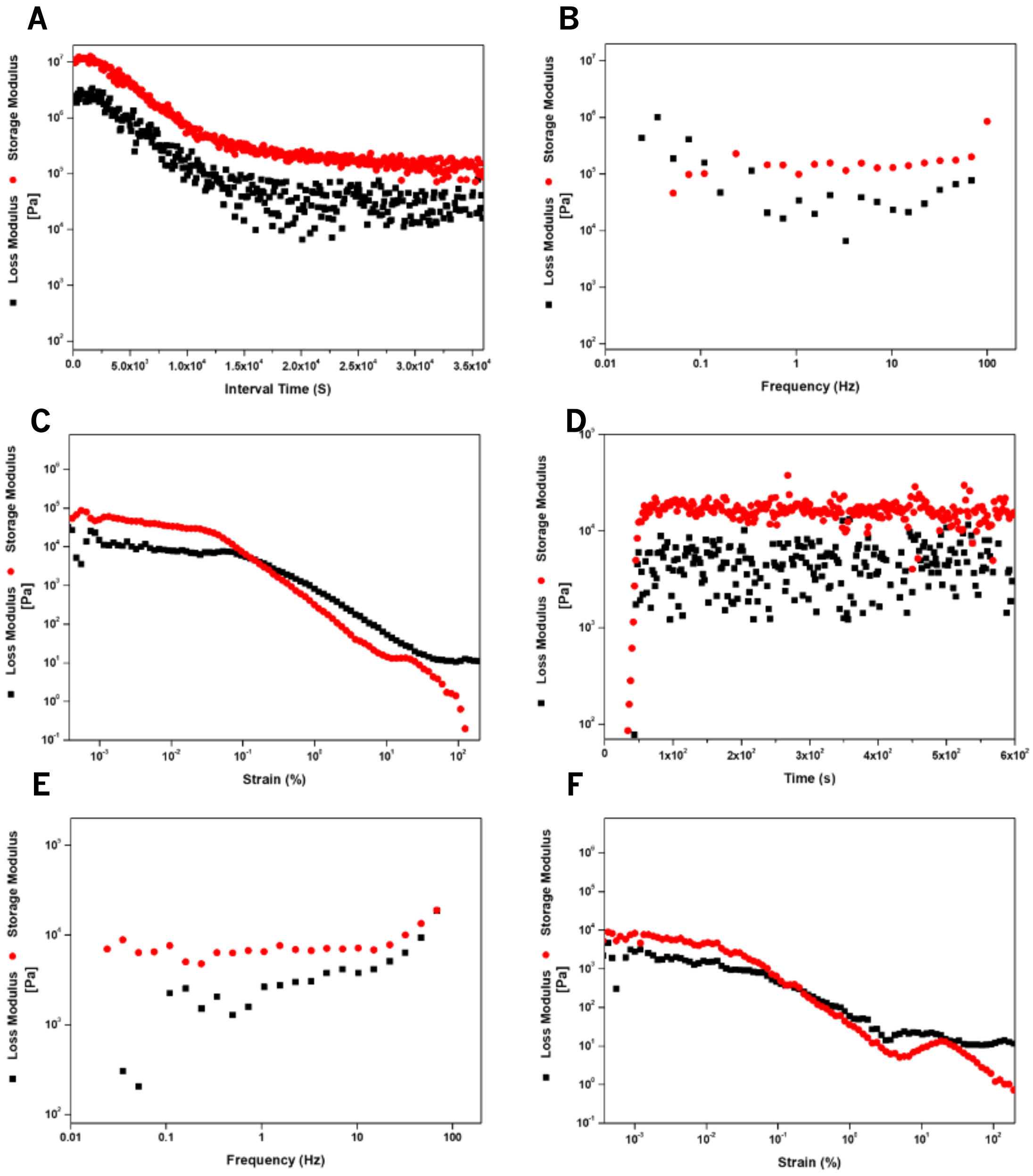

Figure S17. Data for characterisation of hydrogel **18** (0.6wt%) : A- Kinetic study at constant frequency (f =1 Hz) and strain (1x10^{-4} %); B - Mechanical frequency sweep, at constant strain (1x10^{-4} %); C- Large amplitude oscillatory shear(Strain sweep) at constant frequency (f =1 Hz); D- Hydrogel kinetics reformation study at constant frequency (f =1 Hz) and strain (1x10^{-4} %); E- Mechanical frequency sweep of reformed hydrogel , at constant strain (1x10^{-3} %); F- Large amplitude oscillatory shear(Strain sweep) for reformed hydrogel at constant frequency (f =1 Hz).

5. Synthesis

5.1. Synthesis of compound **4**

Compound **1** (4.01 g, 9.07 mmol) was dissolved in dry MeCN (10 mL). Boc_2O (2.38 g, 10.90 mmol) and DMAP (133 mg, 1.09 mmol) were added. The mixture was stirred with a calcium chloride tube. The reaction progress was monitored using ^1H NMR. After the disappearance of the starting material was observed, TMG (2% v/v, 0.2 mL) was added. Once all the intermediates had reacted, the solvent was removed, and the residue was dissolved in ethyl acetate (50 mL). The organic phase was washed with $KHSO_4$ 1M, $NaHCO_3$ 1M, and brine (3x30 mL each). The organic phase was dried with anhydrous magnesium sulfate, and the solvent was removed under reduced pressure. Compound *4* was obtained in 81% yield (3.13 g, 7.37 mmol).

^1H NMR (400 MHz, DMSO) δ 1.31 (s, 9H, $C(CH_3)_3$), 2.76-2.82(dd, J=10.8Hz e J=14Hz, 1H, βCH), 3.02-3.06 (dd, J=3.6Hz e J=13.6Hz, 1H, βCH), 3.69 (s, 3H, -OMe), 4.31-4.37 (m, 1H, αCH), 7.07-7.39 (m, 10H , 8 ArH + NH Boc + CH ΔPhe), 7.38-7.69 (m, 2H,Ar), 9.80 (s, 1H, NH ΔPhe) ppm.

5.2. Synthesis of compound **5**

Compound **2** (2.04 g, 4.47 mmol) was dissolved in dry MeCN (10 mL). Boc_2O (1.17 g; 5.36 mmol) and DMAP (66 mg, 0.54 mmol) were added. The mixture was stirred with a calcium chloride tube. The reaction progress was monitored using ^1H NMR. After the disappearance of the starting material was observed, TMG (2% v/v, 0.2 mL) was added. Once all the intermediates had reacted, the solvent was removed, and the residue was dissolved in ethyl acetate (50 mL). The organic phase was washed with $KHSO_4$ 1M, $NaHCO_3$ 1M, and brine (3x30 mL each). The organic phase was dried with anhydrous magnesium sulfate, and the solvent was removed under reduced pressure. Compound **5** was obtained in 88% yield (1.73 g, 3.95 mmol).

^1H NMR (400 MHz, DMSO) δ 1.42 (s, 9H, $C(CH_3)_3$), 1.81-2.01 (m, 2H, $βCH_2$), 2.56 –2.79 (m, 2H,$γCH_2$), 3.68 (s, 3H, -OMe), 4.03-4.11 (m, 1H,αCH), 7.15 – 737 (m, 10H, 8x ArH, CH ΔPhe, + NH), 7.71 (dd, J = 6.8, 2.9 Hz, 2H, ArH ΔPhe), 9.65 (s, 1H, NH ΔPhe) ppm.

^{13}C NMR (101 MHz, DMSO) δ 28.23 (CH3), 31.51 (Cγ), 33.31 (Cβ), 52.12 (-OMe), 54.26 (Cα), 78.14 (-$C(CH_3)_3$), 125.79 (CH Ar), 126.00 (C ΔPhe), 128.29 (CH Ar), 128.31(CH Ar), 128.45 (CH Ar), 129.44 (CH Ar), 130.21 (CH Ar ΔPhe), 132.02 (CH Ar ΔPhe), 133.27 (C Ar), 141.57 (C Ar), 155.52 (C=O), 165.35 (C=O), 172.46(C=O) ppm.

5.3. Synthesis of compound **6**

Compound *3* (1.53 g, 3.35 mmol) was dissolved in dry MeCN (5 mL). Boc_2O (878 mg; 4.02 mmol) and DMAP (50 mg, 0.40 mmol) were added. The mixture was stirred with a calcium chloride tube. The reaction progress was monitored using ^1H NMR. After the disappearance of the starting material was observed, TMG (2% v/v, 0.1 mL) was added. Once all the intermediates had reacted, the solvent was removed, and the residue was dissolved in ethyl acetate (50 mL). The organic phase was washed with $KHSO_4$ 1M, $NaHCO_3$ 1M, and brine (3x30 mL each). The organic phase was dried with anhydrous magnesium sulfate, and the solvent was removed under reduced pressure. Compound **6** was obtained in 79% yield (1.16 g, 2.65 mmol).

^{1}H NMR (400 MHz, DMSO) δ 1.42 (s, 9H, C(CH$_3$)$_3$), 1.81 – 2.01 (m, 2H, βCH$_2$), 2.58 – 2.75 (m, 2H, γCH$_2$), 3.68 (s, 3H, -OMe), 4.06 – 4.11 (m, 1H, αCH), 7.14-7.38 (m, 10H, 8x ArH, CH ΔPhe , + NH), 7.71 (dd, J = 6.7, 2.9 Hz, 2H, ArH ΔPhe), 9.65 (s, 1H, NH ΔPhe) ppm.

^{13}C NMR (101 MHz, DMSO) δ 28.23 (CH$_3$), 31.51 (Cγ), 33.32 (Cβ), 52.13 (-OMe), 54.26 (Cα), 78.14 (-C(CH$_3$)$_3$), 125.80 (CH Ar), 126.01 (C ΔPhe), 128.24 (CH Ar), 128.30 (CH Ar), 128.32 (CH Ar), 128.46 (CH Ar), 129.45 (CH Ar), 130.22 (CH Ar), 132.03 (CH ArH ΔPhe), 133.28 (C Ar Phe), 141.58 (C Ar Hph), 155.53 (C=O), 165.35 (C=O ΔPhe), 172.47(C=O Hph) ppm.

5.4. Synthesis of compound 7

Compound **5** (1.65 g, 3.76 mmol) was dissolved in TFA (2 mL) during 1h. Ethyl ether was added, and the solvent was evaporated under reduced pressure. Then was added Boc-L-Phenylalanine (998 mg, 3.76 mmol), HBTU (1.57 g, 4.14 mmol) and Et$_3$N (1.58 mL, 11.3 mmol). The reaction was left stirring at room temperature overnight. After complete evaporation, the residue was dissolved in ethyl acetate (100 mL). The organic phase was washed with KHSO$_4$ 1M, NaHCO$_3$ 1M, and brine (3x30 mL each). The organic phase was dried with anhydrous magnesium sulfate, and the solvent was removed under reduced pressure. Compound **7** was obtained in 74% yield (1.74 g, 2.78 mmol).

^{1}H NMR (400 MHz, DMSO) δ 1.31 (s, 9H, C(CH$_3$)$_3$), 1.51 – 1.73 (m, 2H, βCH Hph), 2.19 – 2.41 (m, 2H, γCH$_2$ Hph), 2.83 (dd, J = 13.9, 10.7 Hz, 1H, βCH Phe), 3.17 (dd, J = 13.9, 4.1 Hz, 1H, βCH Phe), 3.72 (s, 3H, -OMe), 3.93-4.05 (m, αCH Hph), 4.65 – 4.73 (m, 1H, αCH Phe), 6.94 (d, J = 7.5 Hz, 1H, NH Hph), 7.00 – 7.42 (m, 14H, 13x ArH + CH ΔPhe), 7.63 – 7.66 (m, 2H, ArH ΔPhe), 8.34 (d, J = 8.5 Hz, 1H, NH Hph), 9.72 (s, 1H, NH ΔPhe) ppm.

^{13C} NMR (101 MHz, DMSO) δ 28.09 (CH$_3$), 31.28 (Cγ Hph) , 33.84 (Cβ Hph), 36.82 (Cβ Phe), 52.20 (-OMe) , 54.07 (Cα Phe), 54.16 (CαHph), 78.15 (-C(CH$_3$)$_3$), 125.59 (CH Ar), 125.70(CH Ar), 126.30 (C ΔPhe), 128.02 (CH Ar), 128.17, (CH Ar) 128.20 (CH Ar), 128.58 (CH Ar), 129.18 (C Ar Phe), 129.46 (CH Ar), 130.16 (CH Ar ΔPhe), 132.52 (CH Ar), 133.16 (C Ar), 137.74 (C Ar), 141.43 (C Ar), 155.38 (C=O), 165.29 (C=O), 171.17 (C=O), 172.12 (C=O) ppm.

5.5. Synthesis of compound 8

Compound **6** (1.16 g, 2.64 mmol) was dissolved in TFA (2 mL) during 1h. Ethyl ether was added, and the solvent was evaporated under reduced pressure. Then was added Boc-*L*-phenylalanine (772 mg, 2.91 mmol), HBTU (1.1 g, 2.91 mmol) and Et$_3$N (1.22 mL, 8.73 mmol). The reaction was left stirring at room temperature overnight. After complete evaporation, the residue was dissolved in ethyl acetate (100 mL). The organic phase was washed with KHSO$_4$ 1M, NaHCO$_3$ 1M, and brine (3x30 mL each). The organic phase was dried with anhydrous magnesium sulfate, and the solvent was removed under reduced pressure. Compound **8** was obtained in 97% yield (1.50 g, 2.56 mmol).

^{1}H NMR (400 MHz, DMSO) δ 1.29 (s, 9H, C(CH$_3$)$_3$), 1.89 – 1.98 (m, 2H, βCH$_2$ Hph), 2.66 –2.70 (m, 2H, γCH$_2$ Hph), 2.73 – 2.80 (m, 1H, βCH Phe), 3.02 (dd, J = 13.9, 3.9 Hz, 1H,βCH Phe), 3.70 (s, 3H, -OMe), 4.24 – 4.30 (m, 1H, αCH Phe)), 4.41 - 4.47 (m, 1H, αCH Phe), 7.03 (d, J = 8.6 Hz, 1H), NH Boc), 7.14 – 7.40 (m, 14H, 13x ArH, CH ΔPhe), 7.65 – 7.68 (m, 2H, 2x ArH ΔPhe), 8.20 (d, J = 7.6 Hz, 1H), 9.70 (s, 1H, NH ΔPhe) ppm.

^{13}C NMR (101 MHz, DMSO) δ 28.11 (CH_3), 30.99 (CγHph), 33.32 (Cβ Hph), 37.21 (Cβ Phe), 52.19 (-OMe), 52.30(Cα Hph), 55.70 (Cα Phe), 78.06 (-C(CH_3)$_3$), 125.85 (CH Ar), 125.90 (C), 126.14 (CH Ar), 127.99 (CH Ar) , 128.33 (CH Ar), 128.56 (CH Ar), 129.18 (CH Ar), 129.48 (CH Ar), 130.07 (CH Ar ΔPhe), 132.21 (CH Ar), 133.21 (C Ar), 138.26(C Ar), 141.43(C Ar), 155.36 (C=O), 165.25 (C=O), 171.55 (C=O), 172.00 (C=O) ppm.

5.6. *Synthesis of compound* ***9***

Compound **4** (1.50 g, 3.83 mmol) was dissolved in TFA (3 mL) during 1h. Ethyl ether was added and the solvent was evaporated under reduced pressure. Then was added Boc-*D*-homophenylalanine (986 mg, 3.53 mmol), HBTU (1.48 g, 3.89 mmol) and Et_3N (1.5 mL, 10.60 mmol). The reaction was left stirring at room temperature overnight. After complete evaporation, the residue was dissolved in ethyl acetate (100 mL). The organic phase was washed with $KHSO_4$ 1M, $NaHCO_3$ 1M, and brine (3x30 mL each). The organic phase was dried with anhydrous magnesium sulfate, and the solvent was removed under reduced pressure. Compound **7** was obtained in 70% yield (1.57 g, 2.68 mmol).

^{1}H NMR (400 MHz, DMSO) δ 1.24 (s, 9H, C(CH_3)$_3$), 1.81 – 2.05 (m, 2H, βCH_2 Hph), 2.45 –2.52 (m, 2H, γCH_2 Hph), 2.83 (dd, J = 13.6, 9.0 Hz, 1H, βCH Phe), 2.99 (dd, J = 13.6, 5.9 Hz, 1H, βCH Phe), 3.70 (s, 3H, -OMe), 4.26 – 4.38 (m, 2H, 2x αCH), 7.05 (d, J = 7.7 Hz, 1H, NH Boc) 7.12 – 7.40 (m, 14H, 13x ArH, CH ΔPhe), 7.65 – 7.68 (m, 2H, 2x ArH ΔPhe), 8.38 (d, J = 7.8 Hz, 1H, NH Hph) 9.60 (s, 1H, NH ΔPhe) ppm.

^{13}C NMR (101 MHz, DMSO) δ 28.02 (CH_3), 31.07 (Cγ), 33.33(Cβ), 37.66 (Cβ), 52.18 (-OMe), 52.31(Cα Hph), 55.86 (Cα Phe), 78.17 (-C(CH3)3), 125.60 (C ΔPhe), 125.83 (CH Ar), 126.23 (CH Ar), 128.03 (CH Ar), 128.23 (CH Ar), 128.33 (CH Ar), 128.39 (CH Ar), 128.55 (CH Ar), 128.63 (CH Ar), 129.26 (CH Ar), 129.47 (CH Ar), 130.20 (CH Ar ΔPhe), 132.69 (CH Ar), 133.16 (C Ar), 137.76 (C Ar), 141.35 (C Ar), 155.34 (C=O), 165.23 (C=O), 171.40 (C=O), 171.83 (C=O) ppm.

5.7. *Synthesis of compound* ***10***

Compound **4** (1.20 g, 2.83 mmol) was dissolved in TFA (2 mL) during 1h. Ethyl ether was added and the solvent was evaporated under reduced pressure. Then was added Boc-*L*-homophenylalanine (790 mg, 2.83 mmol), HBTU (1.18 g, 3.11 mmol) and Et_3N (1.18 mL, 8.5 mmol). The reaction was left stirring at room temperature overnight. After complete evaporation, the residue was dissolved in ethyl acetate (100mL). The organic phase was washed with $KHSO_4$ 1M, $NaHCO_3$ 1M, and brine (3x30 mL each). The organic phase was dried with anhydrous magnesium sulfate, and the solvent was removed under reduced pressure. Compound **10** was obtained in 75% yield (1.25 g, 2.13 mmol).

^{1}H NMR (400 MHz, DMSO) δ 1.36 (s, 9H, C(CH_3)$_3$), 1.65 – 1.82 (m, 2H, βCH_2 Hph), 2.40 – 2.46 (m, 2H, γCH_2 Hph), 2.86 (dd, J = 14.0, 9.5 Hz, 1H, βCH Phe), 3.11 (dd, J = 14.0, 4.7 Hz, 1H, βCH Phe), 3.65 (s, 3H, -OMe), 3.91-3.97 (m, 1H, αCH Hph), 4.71-4.77 (m, 1H, αCH Phe), 6.97 (d, J = 8.2 Hz, 1H, NH Hph), 7.09 – 7.36 (m, 14H, 13x ArH + CH ΔPhe), 7.53 – 7.61 (m, 2H, ArH ΔPhe), 8.05 (d, J = 8.2 Hz, 1H, NH Hph), 9.87 (s, 1H, NH ΔPhe) ppm.

^{13}C NMR (101 MHz, DMSO) δ 28.16 (CH_3), 31.48 (Cβ Hph) , 33.84 (Cγ Hph), 37.12 (Cβ Phe), 52.10 (-OMe) , 53.47 (Cα Phe), 54.06 (Cα Hph), 78.11 (-C(CH_3)$_3$), 125.69(CH Ar), 125.78(C Ar), 126.31 (CH ΔPhe), 128.03 (CH Ar), 128.18, (CH Ar) 128.26 (CH Ar), 128.53 (CH Ar), 129.21 (CH Ar),

129.36 (CH Ar), 129.98 (CH Ar), 131.98 (CH Ar), 133.13 (C Ar), 137.55 (C Ar), 141.54 (C Ar), 155.24 (C=O), 165.27 (C=O), 171.21 (C=O), 171.92 (C=O) ppm.

5.8. *Synthesis of compound **11***

Compound **9** (1.49 g, 2.54 mmol) was dissolved in TFA (3 mL) during 1h. Ethyl ether was added, and the solvent was removed under reduced pressure. Then the residue was dissolved in pyridine (10 mL) and the succinic anhydride (751 mg, 7.5 mmol) was added. The solution was kept under magnetic stirring, at room temperature and under a N_2 atmosphere overnight. Ethyl ether (100 mL) was added, and a precipitate started to form. After filtration, the precipitate was resuspended in HCl (0.1 M) (50 mL) and left stirring for 30 min. The solid was filtered and compound **11** was obtained in a 70% yield (1.04g, 1.77 mmol).

^{1}H NMR (400 MHz, DMSO) δ 1.51 – 1.76 (m, 2H, βCH_2 Hph), 2.10 – 2.45 (m, 6H, γCH_2Hph + 2x CH_2 Suc), 2.83 (dd, J = 13.8, 11.0 Hz, 1H, βCH Phe), 3.19 (dd, J = 13.9, 3.9 Hz, 1H, βCH Phe), 3.72 (s, 3H, - OMe), 4.21-4.28 (m, 1H, αCH Hph), 4.66-4.72 (m, 1H, αCH Phe), 6.97 – 7.06 (m, 2H, CH Ar Hph), 7.08 – 7.42 (m, 12H, 11x CH Ar + CH ΔPhe), 7.62 – 7.66 (m, 2H,CH Ar ΔPhe), 8.06 (d, J = 7.5 Hz, 1H, NH Hph), 8.43 (d, J = 8.5 Hz, 1H, NH Phe), 9.77 (s, 1H, NH ΔPhe) ppm.

^{13}C NMR (101 MHz, DMSO) δ 28.95 (CH_2 Suc), 29.77 (CH_2 Suc),31.10 (Cβ Hph), 33.97(Cγ Hph), 36.78 (Cβ Phe),52.21 (-OMe), 52.68 (Cα Hph), 54.14 (Cα Phe), 125.70 (CH Ar), 125.78 (C ΔPhe),126.30 (CH Ar),128.02 (CH Ar), 128.17 (CH Ar), 128.19 (CH Ar), 128.56 (CH Ar), 129.21 (CH Ar), 129.48 (CH Ar), 130.13 (CH Ar ΔPhe), 132.23 (CH ΔPhe), 133.14 (C Ar ΔPhe), 137.85 (CH Ar Phe), 141.40 (CH Ar Hph), 165.28 (C=O), 171.21(C=O), 171.36(C=O), 171.75(C=O), 173.79 (C=O CO_2H) ppm.

Anal. calcd for $C_{33}H_{35}N_3O_7$(585): calcd: C, 67.68; H, 6.02; N, 7.18; found: C, 67.68; H, 6.20; N, 7.03.

5.9. *Synthesis of compound **12***

Compound **10** (1.25 g, 2.13 mmol) was dissolved in TFA (3 mL) during 1h. Ethyl ether was added, and the solvent was removed under reduced pressure. Then the residue was dissolved in pyridine (10 mL) and the succinic anhydride (641 mg, 6.4 mmol) was added. The solution was kept under magnetic stirring, at room temperature and under a N_2 atmosphere overnight. Ethyl ether (100 mL) was added, and a precipitate started to form. After filtration, the precipitate was suspended in HCl (0.1 M) (50 mL) and left stirring for 30 min. The solid was filtered and compound **12** was obtained in 64% yield (798 mg, 1.36 mmol).

^{1}H NMR (400 MHz, DMSO) δ 1.68 – 1.91 (m, 2H, βCH_2 Hph), 2.30 – 2.47 (m, 6H, γCH_2 Hph + 2x CH_2 Suc), 2.88 (dd, J = 14.0, 9.8 Hz, 1H, βCH Phe), 3.12 (dd, J = 14.0, 4.6 Hz, 1H, βCH Phe), 3.67 (s, 3H, - OMe), 4.26-4.31 (m, 1H, αCH Hph), 4.66-4.71 (m, 1H, αCH Phe), 7.10 – 7.35 (m, 14H, 13x CH Ar + CH ΔPhe), 7.58 – 7.63 (m, 2H,CH Ar ΔPhe), 8.07 (d, J = 8.0 Hz, 1H, NH Hph), 8.13 (d, J = 8.2 Hz, 1H, NH Phe), 9.81 (s, 1H, NH ΔPhe), 12.09 (s, 1H, -CO2H Suc) ppm.

^{13}C NMR (101 MHz, DMSO) δ 29.12 (CH_2 suc), 29.93 (CH_2 Suc),31.29 (Cβ Hph), 34.14 (Cγ Hph), 36.80 (Cβ Phe),52.13 (-OMe), 52.17 (Cα Hph), 54.05 (Cα Phe), 125.70(CH Ar), 125.83 (C ΔPhe),126.35 (CH Ar),128.09 (CH Ar), 128.21 (CH Ar), 128.25 (CH Ar), 128.53 (CH Ar), 129.18 (CH Ar), 129.38 (CH Ar), 130.03 (CH Ar ΔPhe), 131.88 (CH ΔPhe), 133.15 (C Ar ΔPhe), 137.69 (CH Ar Phe), 141.59 (CH Ar Hph), 165.30 (C=O), 171.22(C=O), 171.26(C=O), 171.66 (C=O), 173.90 (C=O CO_2H) ppm.

HRMS (ESI) m/z: [M+H]+ calcd for $C_{33}H_{36}N_3O_7$ 586.66500; found 586.25477.

5.10. *Synthesis of compound* ***13***

Compound **7** (1.66 g, 2.8 mmol) was dissolved in TFA (3 mL) during 1h. Ethyl ether was added, and the solvent was removed under reduced pressure. Then the residue was dissolved in pyridine (10 mL) and the succinic anhydride (841 mg, 8.4 mmol) was added. The solution was kept under magnetic stirring, at room temperature and under a N_2 atmosphere overnight. Ethyl ether (100 mL) was added, and a precipitate started to form. After filtration, the precipitate was suspended in HCl (0.1 M) (50 mL) and left stirring for 30 min. The solid was filtered and compound **13** was obtained in 67% yield (934 mg, 1.59 mmol).

^{1}H NMR (400 MHz, DMSO) δ 1.75-2.03 (m, 2H, βCH_2 Hph), 2.10-2.32 (m, 4H, 2x CH_2 Suc), 2.33-2.45 (m, 2H, γCH_2 Hph), 2.85 (dd, J = 13.5, 8.3 Hz, 1H, βCH_2 Phe), 3.00 (dd, J = 13.5, 6.7 Hz, 1H, βCH Phe), 3.68 (s, 3H, -OMe), 4.26-4.31 (m, 1H, αCH Hph), 4.55-4.61 (m, 1H, αCH Phe), 7.05-7.41 (m, 16H, 15x CH Ar + CH ΔPhe), 8.24 (d, J = 7.3 Hz, 1H, NH Phe), 8.45 (d, J = 7.9 Hz, 1H, NH Hph), 9.58 (d, J = 1.3 Hz, 1H, NH ΔPhe) ppm.

^{13}C NMR (101 MHz, DMSO) δ 28.86 (CH_2 Suc), 29.74 (CH_2 Suc), 31.16(Cβ Hph), 33.19 (Cγ Hph), 37.79(Cβ Phe), 52.18(-OMe), 52.35 (Cα Hph), 54.43 (Cα Phe), 125.82 (CH Ar), 126.32 (CH Ar), 128.07 (CH Ar), 128.30 (CH Ar), 128.32 (CH Ar), 128.55 (CH Ar), 129.24 (CH Ar), 129.50 (CH Ar), 130.20 (CH Ar), 132.45 (CH ΔPhe), 133.13 (C Ar ΔPhe), 137.48 (C Ar), 141.38 (C Ar ΔPhe), 165.22 (C=O ΔPhe), 171.30 (C=O), 171.45 (C=O), 171.70 (C=O), 173.72 (C=O CO_2H) ppm.

Anal. calcd for $C_{33}H_{35}N_3O_7$(585): calcd: C, 67.68; H, 6.02; N, 7.18; found: C, 68.08; H, 6.21; N, 7.13.

5.11. *Synthesis of compound* ***14***

Compound **8** (1.26 g, 2.15 mmol) was dissolved in TFA (3 mL) during 1h. Ethyl ether was added, and the solvent was removed under reduced pressure. Then the residue was dissolved in pyridine (10 mL) and the succinic anhydride (646 mg, 6.45 mmol) was added. The solution was kept under magnetic stirring, at room temperature and under a N_2 atmosphere overnight. Ethyl ether (100 mL) was added, and a precipitate started to form. After filtration, the precipitate was suspended in HCl (0.1 M) (50 mL) and left stirring for 30 min. The solid was filtered and compound **14** was obtained in 78% yield (979 mg, 1.67 mmol).

^{1}H NMR (400 MHz, DMSO) δ 1.89-2.10 (m, 2H, βCH_2 Hph), 2.24-2.37 (m, 4H, 2x CH_2 Suc), 2.61-2.71 (m, 2H, γCH_2 Hph), 2.78 (dd, J = 14.0, 10.1 Hz, 1H, βCH2 Phe), 3.07 (dd, J = 14.0, 4.1 Hz, 1H, βCH Phe), 3.71 (s, 3H, -OMe), 4.38-4.43 (m, 1H, αCH Hph), 4.56-4.63 (m, 1H), αCH Phe), 7.14-7.41 (m, 14H, 13x CH Ar + CH ΔPhe), 7.68 (m, 2H, 2x CH ΔPhe), 8.17 (d, J = 8.3 Hz, 1H, NH Phe), 8.29 (d, J = 7.5 Hz, 1H, NH Hph), 9.68 (d, J = 1.3 Hz, 1H, NH ΔPhe), 12.05 (s, 1H, -CO2H Suc) ppm.

^{13}C NMR (101 MHz, DMSO) δ 29.11 (CH_2 Suc), 29.7 (CH_2 Suc), 31.12 (Cβ Hph), 33.40 (Cγ Hph), 37.39 (Cβ Phe), 52.20 (-OMe), 52.59 (Cα Hph), 53.82 (Cα Phe), 125.87 (CH Ar), 125.98 (CH Ar), 126.18 (C Ar) 127.99 (CH Ar), 128.31 (CH Ar), 128.38 (CH Ar), 128.56 (CH Ar), 129.16 (CH Ar), 129.86 (CH Ar Phe), 132.07 (CH Ar), 133.25 (C Ar), 138.04 (C Ar),141.44 (C Ar), 165.28 (C=O), 171.57 (C=O), 171.57 (C=O), 171.59 (C=O), 173.76 (C=O CO_2H) ppm.

Anal. calcd for $C_{33}H_{35}N_3O_7$(585): calcd: C, 67.68; H, 6.02; N, 7.18; found: C, 68.00; H, 6.31; N, 6.95.

5.12. *Synthesis of compound **15***

Compound **11** (600 mg, 1.02 mmol) was dissolved in 1,4-dioxane (10 mL) and NaOH (1M) (3 mL). The reaction was monitored by TLC. When the starting material was consumed, the organic solvent was removed under reduced pressure and the reaction mixture was acidified to pH 3 with $KHSO_4$ (1M). A solid precipitate was filtered, to afford compound **15** in 87% yield (509 mg, 0.89 mmol).

^{1}H NMR (400 MHz, DMSO) δ 1.53 – 1.70 (m, 2H, βCH_2 Hph), 2.15 – 2.47 (m, 6H, γCH_2 Hph + 2x CH_2 Suc), 2.82 (dd, J = 13.8, 11.3 Hz, 1H, βCH Phe), 3.19 (dd, J = 13.8, 3.5 Hz, 1H, βCH Phe), 4.21-4.27 (m, 1H, αCH Hph), 4.66-4.71 (m, 1H, αCH Phe), 6.99 – 7.02 (m, 2H, CH Ar Hph), 7.09 – 7.38 (m, 12H, 11x CH Ar + CH ΔPhe), 7.58 – 7.63 (m, 2H,CH Ar ΔPhe), 8.05 (d, J = 7.6 Hz, 1H, NH Hph), 8.40 (d, J = 8.6 Hz, 1H, NH Phe), 9.61 (s, 1H, NH ΔPhe) ppm.

^{13}C NMR (101 MHz, DMSO) δ 29.00 (CH_2 Suc), 29.81 (CH_2 Suc), 31.10 (Cβ Hph), 34.00 (Cγ Hph), 36.87 (Cβ Phe), 52.64 (Cα Hph), 54.21 (Cα Phe), 125.71 (CH Ar), 126.27 (C ΔPhe), 126.55 (CH Ar), 128.00 (CH Ar), 128.23 (CH Ar), 128.48 (CH Ar), 129.24 (CH Ar), 130.03 (CH Ar), 131.98 (CH Ar), 133.54 (CH ΔPhe), 137.94 (CH Ar Phe), 141.43 (CH Ar Hph), 166.17 (C=O ΔPhe), 171.02 (C=O), 171.18 (C=O), 171.71 (C=O), 173.83 (C=O CO_2H) ppm.

Anal. calcd for $C_{32}H_{33}N_3O_7$ (571): calcd: C, 67.24; H, 5.82; N, 7.35; found: C, 67.27; H, 6.09; N, 7.15.

5.13. *Synthesis of compound **16***

Compound **12** (398 mg, 0.68 mmol) was dissolved in 1,4-dioxane (4 mL) and NaOH (1M) (3 mL). The reaction was monitored by TLC. When the starting material was consumed, the organic solvent was removed under reduced pressure and the reaction mixture was acidified to pH 3 with $KHSO_4$ (1M). A solid precipitate was filtered, to afford compound **16** in 75% yield (291 mg, 0.51 mmol).

^{1}H NMR (400 MHz, DMSO) δ 1.64 – 1.90 (m, 2H, βCH_2 Hph), 2.13 – 2.47 (m, 6H, γCH_2 Hph + 2x CH_2 Suc), 2.78 - 2.89 (m, 1H, βCH Phe), 3.12 – 3.21 (m, 1H, βCH Phe), 4.21 – 4.29 (m, 1H, αCH Hph), 4.64 – 4.72 (m, 1H, αCH Phe), 6.99 – 7.38 (m, 12H, 13x CH Ar + CH ΔPhe), 7.58 – 7.63 (m, 2H,2x CH Ar ΔPhe), 8.05 (d, J = 7.9 Hz, 1H, NH Hph), 8.09 (d, J = 8.2 Hz, 1H, NH Phe), 9.62 (s, 1H, NH ΔPhe), 12.40 (bs, 2H, -CO2H) ppm.

^{13}C NMR (101 MHz, DMSO) δ 29.14 (CH_2 Suc), 29.96 (CH_2 Suc), 31.29 (Cβ Hph), 34.10 (Cγ Hph), 36.84 (Cβ Phe), 52.19 (Cα Hph), 54.15 (Cα Phe), 125.70 (CH Ar), 126.32 (CH Ar), 126.48 (C Ar), 127.73 (CH Ar), 128.82 (CH Ar), 127.92 (CH Ar), 128.02 (CH Ar), 128.21 (CH Ar), 1280.91 (CH Ar), 129.71 (CH ΔPhe), 131.59 (CH Ar Phe), 131.78 (CH Ar), 133.51 (C), 133.54 (C Ar), 137.82 (C Ar),141.61 (C Ar), 166.16 (C=O), 170.98 (C=O), 171.21 (C=O), 171.65 (C=O), 173.92 (C=O CO_2H) ppm.

HRMS (ESI) m/z: [M+H]+ calcd for $C_{32}H_{34}N_3O_7$ 572.23968; found 572.23906.

5.14. *Synthesis of compound **17***

Compound **13** (500 mg, 0.85 mmol) was dissolved in 1,4-dioxane (10 mL) and NaOH (1M) (3 mL). The reaction was monitored by TLC. When the starting material was consumed, the organic solvent was removed under reduced pressure and the reaction mixture was acidified to pH 3

with $KHSO_4$ (1M). A solid precipitate was filtered, to afford compound **17** in 86% yield (417 mg, 0.73 mmol).

^{1}H NMR (400 MHz, DMSO) δ 1.74-2.05 (m, 2H, , βCH$_2$ Hph), 2.08-2.32 (m, 4H, 2x CH$_2$ Suc), 2.36-2.45 (m, 2H, γCH$_2$ Hph), 2.84 (dd, J = 13.6, 8.5 Hz, 1H, βCH Phe), 3.00 (dd, J = 13.5, 6.6 Hz, 1H, βCH Phe), 4.27-4.32 (m, 1H, αCH Hph), 4.56-4.61 (m, 1H, αCH Phe), 7.09-7.37 (m, 14H, 13x CH Ar + CH ΔPhe),7.60-7.65(m, 2H, CH Ar Phe), 8.22 (d, J = 7.5 Hz, 1H, NH Phe), 8.41 (d, J = 7.9 Hz, 1H, NH Hph), 9.42 (s, 1H, NH ΔPhe) ppm.

^{13}C NMR (101 MHz, DMSO) δ 28.94 (CH$_2$ Suc), 29.82 (CH$_2$ Suc), 31.21(Cβ Hph), 33.46 (Cγ Hph), 37.85 (Cβ Phe), 52.47 (Cα Hph), 54.44 (Cα Phe), 125.82(CH Ar), 126.35 (CH Ar),128.10 (CH Ar), 128.32 (CH Ar), 128.35 (CH Ar), 128.39 (CH Ar), 128.45 (C Ar), 128.51 (CH Ar), 129.20 (C Ar), 129.27 (CH Ar), 130.06 (C Ar), 130.12 (CH Ar), 132.24 (CH ΔPhe), 133.48 (C Ar ΔPhe), 137.56 (C Ar), 141.54 (C Ar ΔPhe), 166.14 (C=O ΔPhe), 171.31 (C=O Suc), 171.45 (C=O Hph), 173.78 (C=O CO_2H) ppm.

Anal. calcd for $C_{32}H_{33}N_3O_7$ (571): calcd: 67.24; H, 5.82; N, 7.35; found: C, 67.16; H, 6.05; N, 7.20.

5.15. *Synthesis of compound **18***

Compound **14** (498 mg, 0.85 mmol) was dissolved in 1,4-dioxane (4 mL) and NaOH (1M) (3 mL). The reaction was monitored by TLC. When the starting material was consumed, the organic solvent was removed under reduced pressure and the reaction mixture was acidified to pH 3 with $KHSO_4$ (1M). A solid precipitate was filtered, to afford compound **18** in 89% yield (426 mg, 0.75 mmol).

^{1}H NMR (400 MHz, DMSO) δ 1.89-2.09 (m, 2H, , βCH$_2$ Hph), 2.23-2.36 (m, 4H, 2x CH$_2$ Suc), 2.60-2.68 (m, 2H, γCH$_2$ Hph), 2.71 - 2.81 (m, 1H, βCH Phe), 3.06 (dd, J = 14.0, 4.1 Hz, 1H, βCH Phe), 4.36-4.42 (m, 1H, αCH Hph), 4.56-4.62 (m, 1H, αCH Phe), 7.04-7.39 (m, 14H, 13x CH Ar + CH ΔPhe), 7.67 (dd, J = 6.8, 3.0 Hz, 2H, CH Ar Phe), 8.18 (d, J = 8.3 Hz, 1H, NH Phe), 8.29 (d, J = 7.5 Hz, 1H, NH Hph), 9.68 (s, 1H, NH ΔPhe), 12.04 (bs, 2H, 2x –CO2H) ppm.

^{13}C NMR (101 MHz, DMSO) δ 29.09 (CH$_2$ Suc), 29.95 (CH$_2$ Suc), 31.20(Cβ Hph), 33.39 (Cγ Hph), 37.37 (Cβ Phe), 52.57 (Cα Hph), 53.80 (Cα Phe), 125.85(CH Ar), 126.16 (CH Ar),127.97 (CH Ar), 128.29 (CH Ar), 128.36 (CH Ar), 128.54 (CH Ar), 129.14 (C Ar), 129.46 (CH Ar), 130.10 (CH Ar), 132.05 (C Ar), 133.23 (C Ar), 138.02 (C Ar), 141.43 (C Ar), 165.27 (C=O), 171.04 (C=O), 171.56 (C=O), 173.74 (C=O CO_2H) ppm.

Anal. calcd for $C_{32}H_{33}N_3O_7$ (571): calcd: 67.24; H, 5.82; N, 7.35; found: C, 67.17; H, 5.83; N, 7.09
